# A Distalless-responsive enhancer of the Hox gene *Sex combs reduced* is required for segment- and sex-specific sensory organ development in *Drosophila*

**DOI:** 10.1371/journal.pgen.1007320

**Published:** 2018-04-10

**Authors:** Sebnem Ece Eksi, Olga Barmina, Christopher L. McCallough, Artyom Kopp, Teresa Vales Orenic

**Affiliations:** 1 Department of Biological Sciences, University of Illinois at Chicago, Chicago, IL, United States of America; 2 Department of Evolution and Ecology, University of California-Davis, Davis, CA, United States of America; Wayne State University, UNITED STATES

## Abstract

Hox genes are involved in the patterning of animal body parts at multiple levels of regulatory hierarchies. Early expression of Hox genes in different domains along the embryonic anterior-posterior (A/P) axis in insects, vertebrates, and other animals establishes segmental or regional identity. However, Hox gene function is also required later in development for the patterning and morphogenesis of limbs and other organs. In *Drosophila*, spatiotemporal modulation of *Sex combs reduced* (*Scr*) expression within the first thoracic (T1) leg underlies the generation of segment- and sex-specific sense organ patterns. High *Scr* expression in defined domains of the T1 leg is required for the development of T1-specific transverse bristle rows in both sexes and sex combs in males, implying that the patterning of segment-specific sense organs involves incorporation of *Scr* into the leg development and sex determination gene networks. We sought to gain insight into this process by identifying the *cis*-and *trans*-regulatory factors that direct *Scr* expression during leg development. We have identified two *cis*-regulatory elements that control spatially modulated *Scr* expression within T1 legs. One of these enhancers directs sexually dimorphic expression and is required for the formation of T1-specific bristle patterns. We show that the Distalless and Engrailed homeodomain transcription factors act through sequences in this enhancer to establish elevated *Scr* expression in spatially defined domains. This enhancer functions to integrate *Scr* into the intrasegmental gene regulatory network, such that *Scr* serves as a link between leg patterning, sex determination, and sensory organ development.

## Author summary

The body plans of most animals include repeated structures, such as limbs, that are similar in structure but have distinguishing features that adapt them for specific functions. Morphological variations among repeated structures are controlled by Hox genes, which exhibit dynamic spatial and temporal expression throughout development. Changes in Hox gene expression are often correlated with the evolution of novel morphologies. Thus, elucidating the spatial regulation of Hox genes is essential for understanding the diversity of animal form and function. We investigated the regulation of the fruit fly Hox gene *Sex combs reduced* (*Scr)*, which is expressed in the first pair of legs (T1) and is required for the development of segment- and sex-specific sensory organs. We identified two genomic regions (enhancers) that control *Scr* expression in T1 legs. One of these enhancers is required for the formation of segment- and sex-specific sense organs, and responds to key regulators, such as Distalless and Engrailed, that establish anterior-posterior and proximo-distal coordinates in the developing leg. Our results suggest that morphological diversity in development and evolution can be achieved by integrating Hox genes into the relatively downstream tiers of gene regulatory networks that control spatial patterning.

## Introduction

Body structures formed at varying positions along the anterior-posterior (A/P) axis of bilaterian embryos acquire distinct morphologies based on the differential expression and function of Hox genes. Subsequently, Hox genes can have narrower post-embryonic functions in the development of specific organs such as the limbs of *Drosophila* and other insects. In *Drosophila*, expression of Hox genes in the limb primordia is often heterogeneous, exhibiting spatiotemporal variations in expression levels that are likely to be critical for generation and patterning of diverse cells types [1–8]. In a few cases, elevated Hox gene expression in defined regions of the *Drosophila* limb primordia at specific stages of development has been correlated with formation of particular cell types and patterns. For example, high-level expression of the Hox gene *Ultrabithorax (Ubx)* in the femur of legs from the second thoracic segment (T2) is necessary for patterning of trichomes, and variation in trichome patterns among various *Drosophila* species is correlated with differential *Ubx* expression [6]. Similarly, elevated expression of *Ubx* and another Hox gene, *Sex combs reduced* (*Scr*), in specific domains of leg discs of the third and first thoracic segments (T3 and T1), respectively, is essential for development of distinct groups of sense organs [9, 10]. This includes male-specific sex combs, which are found in a subset of *Drosophila* species and exhibit strikingly diverse patterns that are associated with variation in *Scr* expression [9, 11]. To determine the mechanisms responsible for spatially modulated Hox gene expression during limb development and understand how these mechanisms evolve, it is necessary to identify the *cis*- and *trans*-regulatory elements that direct Hox gene expression during limb development. Toward this end, we have studied the regulation of *Scr* expression in the T1 leg of *Drosophila melanogaster* from larval through pupal stages of development.

Each leg pair has a stereotypical pattern of small mechanosensory microchaetae, which on the surface of T2 legs are organized into longitudinal bristle rows (LBRs) that extend along the proximal-distal (P/D) axis of the leg and are precisely positioned along the leg circumference. In addition, on T1 and T3 legs, groups of microchaete bristles are organized in rows that are orthogonal to the LBRs, a pattern not observed on T2 legs. These transverse bristle rows (TBRs) are located on the anterior surface of the T1 legs and on the posterior surface of T3 legs [12–14]. Diverse sense organ patterns and other morphological features of the three pairs of *Drosophila* adult legs are specified by the dynamic expression and function of *Scr* and *Ubx* in T1 and T3 legs, respectively. *Scr* and *Ubx* function continuously throughout leg development, beginning in embryogenesis, when the leg primordia consist of small groups of cells in the embryonic ectoderm, continuing throughout larval, prepupal and pupal stages [2–7, 15–21].

In T1 legs, the spatiotemporal pattern of *Scr* expression within the leg primordia varies throughout the course of leg development [4]. Low-level *Scr* expression is observed throughout the T1 leg, and elevated expression can be detected in specific regions, including in the primordia of T1-leg specific sensory organs [9, 10]. We have previously shown that expression of *Scr* and *Ubx* is elevated in the TBR primordia of T1 and T3 legs, respectively. The upregulated expression of these Hox genes leads to the differential expression of Delta, a Notch ligand, resulting in the formation of TBRs instead of LBRs [10, 22]. In T1 legs, upregulated *Scr* expression is observed in the antero-ventral region of the distal half of the tibia and in the first tarsal segment (ta1), marking the TBR primordia. In male pupal legs, *Scr* expression is further elevated, compared to in females, surrounding the primordia of the most distal TBR on ta1. This row forms the male sex comb, which consists of thickened and highly pigmented sex comb bristles (SCBs) and is rotated 90 degrees relative to the TBRs [9]. In this context, *Scr* functions to activate expression of the sex determination gene, *doublesex* (*dsx*), in the sex comb primordia, and subsequently the two genes participate in a regulatory feedback loop that maintains *Scr* and *dsx* expression [23].

In this study, we sought to understand how the dynamic expression of *Scr* is controlled during three distinct stages of leg development, by identifying and functionally characterizing *cis*-regulatory elements that control expression of this gene in the TBR/SCB primordia. We identified two enhancers, one in an intron (intronic enhancer) and the other located upstream of *Scr* (upstream enhancer). Both enhancers direct elevated expression in the TBR primordia of prepupal legs, but only the upstream enhancer directs sexually dimorphic expression of *Scr* in male pupal legs. Deletion of the upstream enhancer results in loss of both TBRs and sex combs. We show that the upstream enhancer exploits the gene network that is responsible for the development of the three-dimensional structure of all legs to establish elevated *Scr* expression in the TBR and SCB primordia. Our studies also identified new regulatory links between *Scr* and the leg patterning transcription factors Distalless (Dll) and Engrailed (En) and have provided new insights into the function of *Scr* in connecting the leg gene regulatory network and pathways that control sense organ development.

## Results

### Two enhancers regulate elevated *Scr* expression in the T1 leg

**Fig 1:**
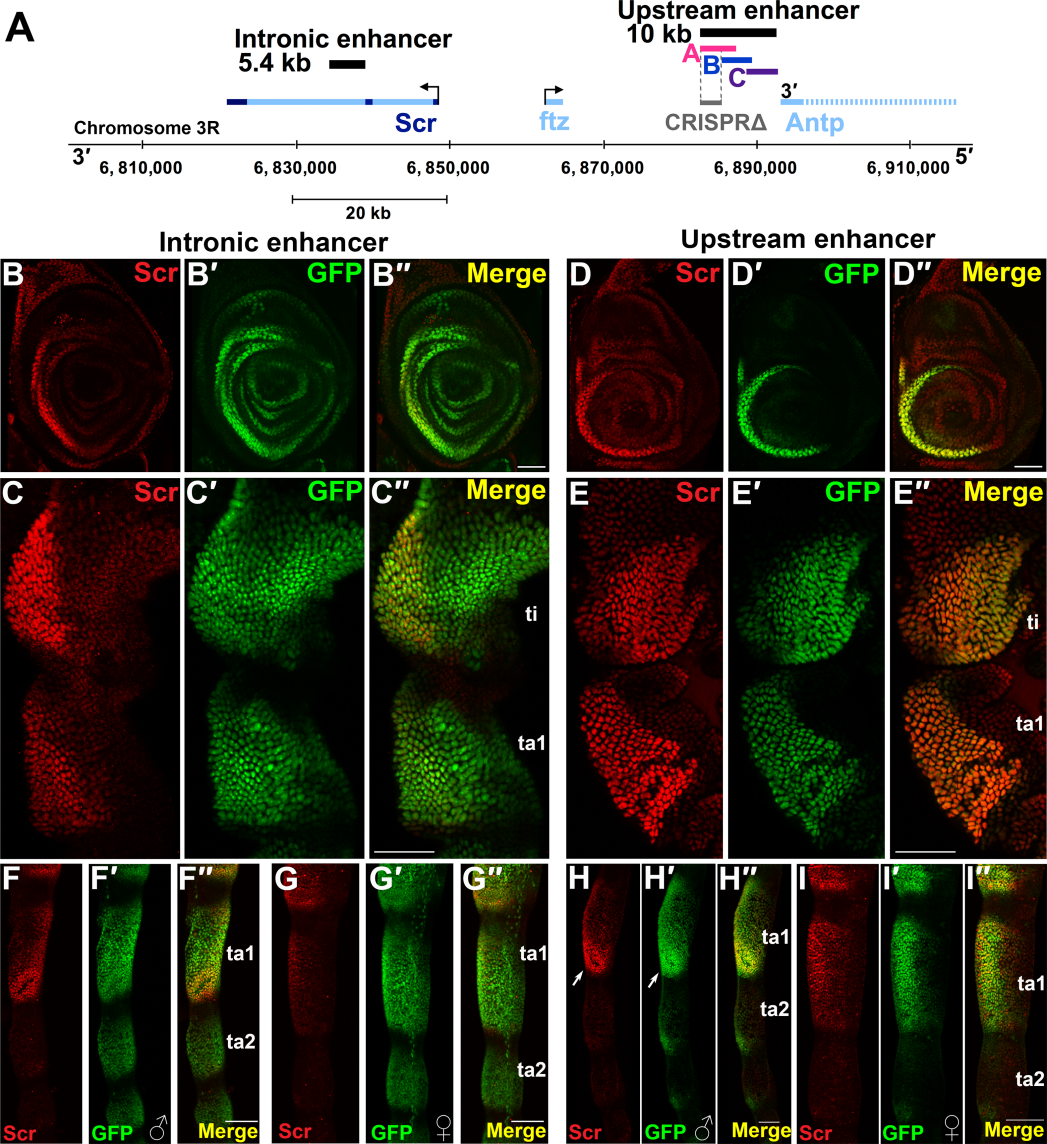
Early *Scr* expression in the TBR primordia is directed by two enhancers, one of which directs sexually dimorphic expression in the SCB primordia of pupal legs. A. Map of the *Scr* region (24). Intronic and upstream enhancers are shown relative to the *Scr* locus (dark blue lines designate exons, and light blue lines designate introns; introns are shown only for Scr). The intronic enhancer is located within the second intron of the *Scr* transcription unit, while the upstream enhancer is situated 33 kb 5’ of the *Scr* transcription start site. Three overlapping subfragments of the upstream 10 kb XbaI fragment, A, B and C were tested for enhancer activity, of which only A directed reporter expression in legs. The gray bar indicates the CRISPR/Cas9 deletion, *Scr*^*ΔCRE(upA-B)*^. Dashed lines indicate the deletion boundaries within fragment A. B-I". Intronic and upstream enhancer reporter gene expression in larval, prepupal and pupal legs. In all images of larval leg discs anterior is to the left and dorsal is up; for prepupal and pupal legs, proximal is up, and antero-ventral aspects are shown. B-C". Reporter expression from the intronic enhancer. GFP expression (green) from the intronic enhancer in 3rd instar larval leg discs (B-B") and in the first tarsal segment (ta1) and tibia (ti) of 6h APF prepupal legs (C-C") compared to endogenous Scr expression (anti-Scr, red). Note that reporter gene expression is expanded relative to the domain of elevated endogenous Scr expression (Size bars = 50μ). D-E". Reporter expression from the upstream enhancer. GFP expression (green) directed by the upstream enhancer (Fragment A) in 3rd instar larval leg discs (D-D") and 6h APF prepupal legs (E-E") compared to endogenous *Scr* expression (red). Note that reporter expression faithfully reproduces endogenous elevated *Scr* expression in the TBR primordia. F-G". Reporter expression from the intronic enhancer in male (F-F") and female (G-G") 24h APF pupal legs. H-I". Reporter expression from the upstream enhancer in male (H-H") and female (I-I") 24h APF pupal legs. Note that the sexually dimorphic elevated Scr expression in male legs around the presumptive SCB primordia (arrows in H and H') is recapitulated by the upstream enhancer.

To better understand how *Scr* levels are modulated within the T1 leg, we sought to identify *cis*-regulatory sequences that direct *Scr* expression in the TBR and SCB primordia. We tested multiple *lacZ* reporters from the *Scr-Antennapedia* (*Antp*) region that had previously been reported to direct tissue-specific reporter expression in embryos [24] and identified two DNA fragments that directed expression in the medial region of the leg imaginal discs in a pattern similar to elevated *Scr* expression in the TBR primordia (Fig 1A). A screen of Janelia Gal4 lines [25, 26] covering the entire *Scr*-*Antp* region did not identify any additional regions that reproduced endogenous *Scr* expression in leg discs and pupal legs, although several fragments drove ectopic leg expression. Putative enhancers near the *Scr* locus were then assayed in site-specific integration GFP reporter plasmids, pS3aG or pS3aG-ScrP; the latter was generated by replacing the basal *hsp70* promoter in pS3aG with a 1.2kb *Scr* promoter fragment that includes a promoter-proximal tethering element [27] (see Methods). Based on observations from Gindhart et al., (1995) and this study (S1 Table), which have shown that some *Scr* enhancers preferentially activate expression from an *Scr* promoter, the pS3aG-ScrP plasmid was used to test the majority of enhancer sequences described throughout this report.

In our initial analyses, we determined that a 5.4 kb fragment from the second intron of *Scr* directs reporter expression that overlaps the domain of elevated *Scr* expression in larval and prepupal legs, although ectopic expression is observed in distal leg segments and along the leg circumference (Fig 1A, 1B and 1C"). A second enhancer located within a 10kb XbaI fragment, which maps 33 kb upstream of the *Scr* locus, has been reported to direct expression in the T1 embryonic segment [24] and in third instar leg discs [28]. This 10kb XbaI fragment overlaps a region predicted via breakpoint mapping to contain an *Scr* leg imaginal disc enhancer [29]. We tested three smaller overlapping fragments, A, B and C (Fig 1A), from the 10kb XbaI fragment and found that, while fragments B and C did not direct reporter expression, fragment A directed expression that faithfully reproduced the elevated *Scr* expression pattern during different stages of leg development (Fig 1D and 1E"). Surprisingly, both the intronic and upstream enhancer fragments direct similar patterns of reporter gene expression in all three leg pairs, as opposed to the T1 leg specific expression of endogenous *Scr* (S1C-C" Fig). This may be due to the integration of reporter genes into sites other than the endogenous *Scr* locus, such that putative *Scr* enhancers are not associated with silencing epigenetic elements that normally block expression in T2 and T3 legs and maintain expression in T1 legs (see Discussion) [4, 29–32].

In addition to the elevated expression observed in the TBR/SCB primordia, *Scr* is expressed at lower levels throughout the T1 leg disc, and several observations suggest that sequences directing this low-level expression are contained within the 1.2 kb *Scr* promoter fragment included in pS3aG-ScrP. We observed differences in the activity of reporters with a heterologous enhancer-promoter system in which the intronic enhancer was in conjunction with the basal *hsp70* promoter (*ScrIntronic-hsp70P-GFP*) vs an *Scr* promoter fragment (*ScrIntronic-ScrP-GFP*) (S1 Fig and S1 Table). The *ScrIntronic-hsp70P-GFP* transgene directs reporter gene expression within the TBR primordia of prepupal legs (S1A-A" Fig) but not the broad low-level expression. On the other hand, the low-level expression of *Scr* in T1 legs was recapitulated by the *ScrIntronic-ScrP-GFP* transgene, suggesting that the *Scr* promoter or proximal sequences direct the low-level expression (S1 Table). Furthermore, the intronic enhancer directs weaker and more uneven expression in the TBR region in conjunction with the *hsp70* promoter compared to *ScrP* (compare S1A'–S1C' Fig), suggesting that sequences in the promoter fragment are necessary for full enhancer activity. To determine whether the broad low-level *Scr* expression is directed by the *Scr* promoter fragment, we generated an *ScrP-GFP* transgenic line without any additional enhancer sequences. Uniform nuclear reporter expression was observed in the leg tissue from transgenic animals that bear this reporter construct (S1B' Fig).

Combined, our observations suggest that at least two separate enhancers direct elevated *Scr* expression in the prepupal leg TBR/SCB primordia, while the *Scr* promoter or promoter proximal sequences direct broad low-level *Scr* expression in T1 legs.

### The upstream enhancer directs sexually dimorphic expression

Although both the intronic and the upstream enhancer drive expression in the TBR primordia of larval and prepupal legs, we observed a significant difference in the activity of the two enhancers in pupal legs between 20-24hrs APF (Fig 1F-I"). It has previously been shown that *Scr* expression is specifically elevated in cells surrounding the SCB primordia of male pupal legs to levels above those observed in the TBR primordia at this stage [9]. We found that the upstream enhancer reproduces this high-level SCB expression in male legs (Fig 1H-I"), while the intronic enhancer fails to do so (Fig 1F-G"). As previously reported, at later stages Scr expression is downregulated in the TBR and SCB bristle precursors [9, 10], and is dispensable for the morphogenesis of bristle shafts [33]. Expression of intronic and upstream reporter genes also exhibits this downregulation, which is evident in the SCB primordia in Fig 1F' and 1H'. Combined, these observations suggest that, while both enhancers direct expression in the TBR primordia, sexually dimorphic expression of *Scr* is activated exclusively by the upstream enhancer, implying functional distinction between the two enhancers.

### The upstream enhancer is required for T1 leg-specific sense organ development

**Fig 2:**
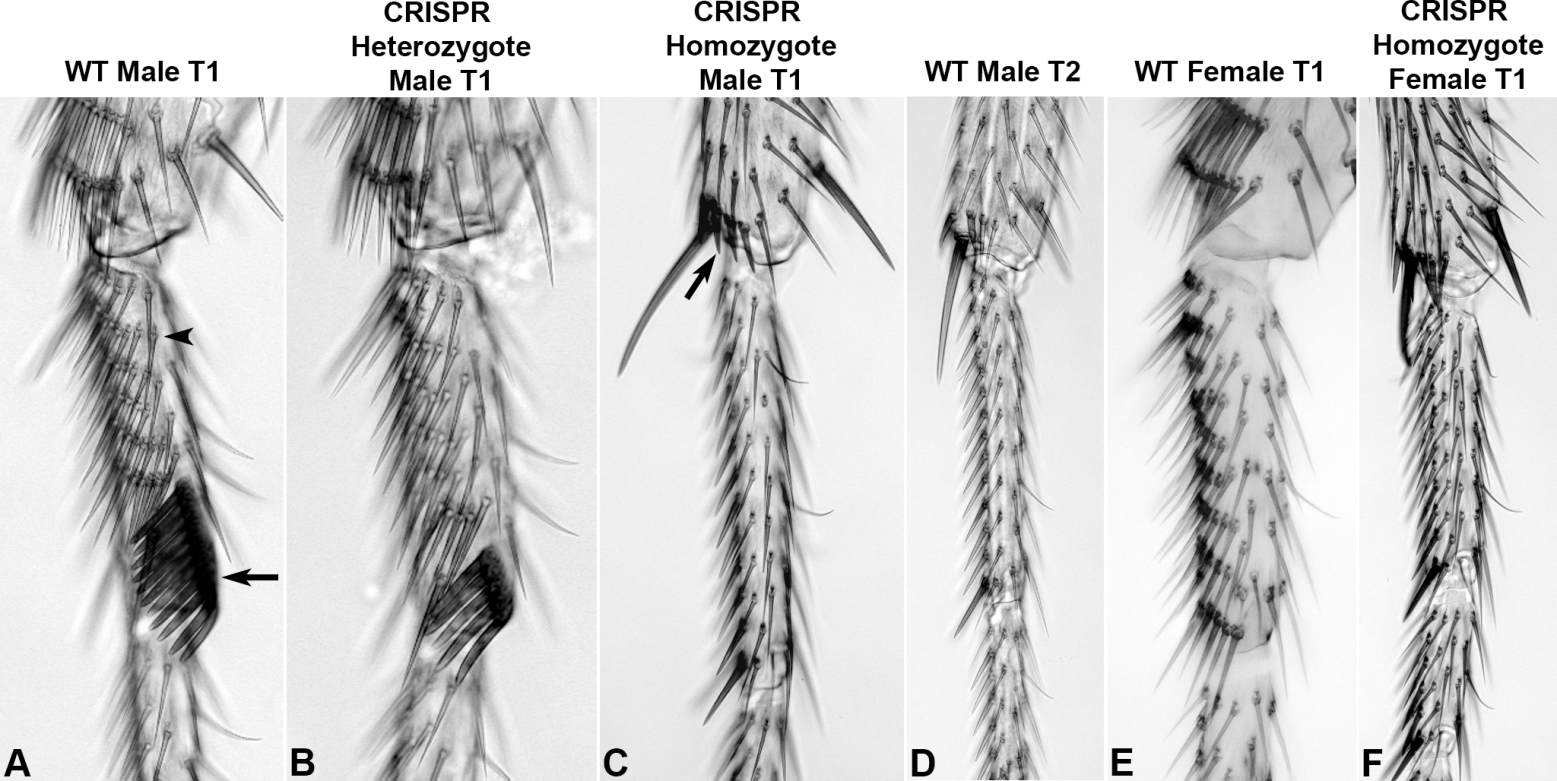
The upstream *Scr* enhancer is required for development of the T1 leg TBRs and SCBs. In all leg images, proximal is up and the ventral surface is to the left. A. Wild-type adult male T1 leg with TBRs (arrowhead designates one TBR) and sex comb (arrow) located on the anterior surface. B. CRISPR deletion of the upstream enhancer results in a reduction of sex comb bristle number in heterozygote males (*Scr*^*ΔCRE(upA-B)*^/+), as observed in legs heterozygous for *Scr* null alleles (29). C. Males homozygote for the CRISPR deletion (*Scr*^*ΔCRE(upA-B)*^/ (*Scr*^*ΔCRE(upA-B)*^/+) completely lack TBRs and SCBs on their T1 legs. Arrow designates ectopic spur bristles typical of T2 legs. D. Stereotypical pattern of sensory organs on the wild-type T2 leg. Note the similarities between the homozygote CRISPR deletion line (C) and a wild-type T2 leg, including the formation of spur bristles on the T1 leg tibia (arrow in C), as observed in legs with reduced *Scr* function [34]. E. Wild-type female T1 leg with TBRs located on the anterior surface. F. Homozygote CRISPR deletion of the upstream enhancer results in loss of TBRs in female T1 legs.

Our findings indicate that the intronic and upstream enhancers share common activities in the TBR, suggesting that these enhancers could have redundant or additive functions in development of T1-specific sense organs. To determine how each enhancer contributes to the development of TBRs and SCBs, we assayed adult leg phenotypes in animals carrying CRISPR/Cas9 deletions of each enhancer. For the upstream enhancer, we deleted a 3763 bp region (Fig 1A) to generate a new *Scr* allele, *Scr*^*ΔCRE(upA-B)*^. The deleted region corresponds to a subfragment of A, A3, which drives similar levels and pattern of expression as does fragment A (see enhancer analysis below). Heterozygous *Scr*^*ΔCRE(upA-B)*^/+ males exhibited a reduction in SCB numbers (Fig 2B) as compared to a wild type T1 leg (Fig 2A), a phenotype similar to that observed in males heterozygous for amorphic alleles of *Scr* [31]. Most homozygous males completely lacked SCBs and TBRs and acquired a bristle pattern like that of wild type T2 legs (compare Fig 2C and 2D). Most females homozygous for *Scr*^*ΔCRE(upA-B)*^ lacked TBRs in the tibia and ta1 segments (Fig 2F), while heterozygous females had reduced numbers of TBR bristles. However, vestigial TBRs were observed in the distal tibia in a few homozygous males and females, suggesting that the activity of the intronic enhancer might contribute to the formation of TBRs in the tibia. Homozygous adults carrying the deletion had reduced viability and usually had to be rescued from their pupal cases. However, other than the T1 leg phenotype, no obvious morphological defects were observed. In particular, the morphology of T2 and T3 legs was normal, including the presence of TBRs in the T3 leg (S2A and S2B Fig).

Flies homozygous for a 5.7 kb deletion of the intronic enhancer (gift from J.A. Kennison and M.T. Cooper, pers. comm.) did not exhibit any TBR or sex comb phenotypes, suggesting that the intronic enhancer is not essential for development of these structures. Combined, our observations indicate that the upstream enhancer is essential for development of TBRs and SCBs, and sufficient to direct necessary levels of *Scr* expression in the tarsus in the absence of intronic enhancer function. We therefore focused further analyses on the upstream enhancer.

### Localized activation and stage-specific repression of *Scr* are regulated by multiple regions of the upstream enhancer

**Fig 3:**
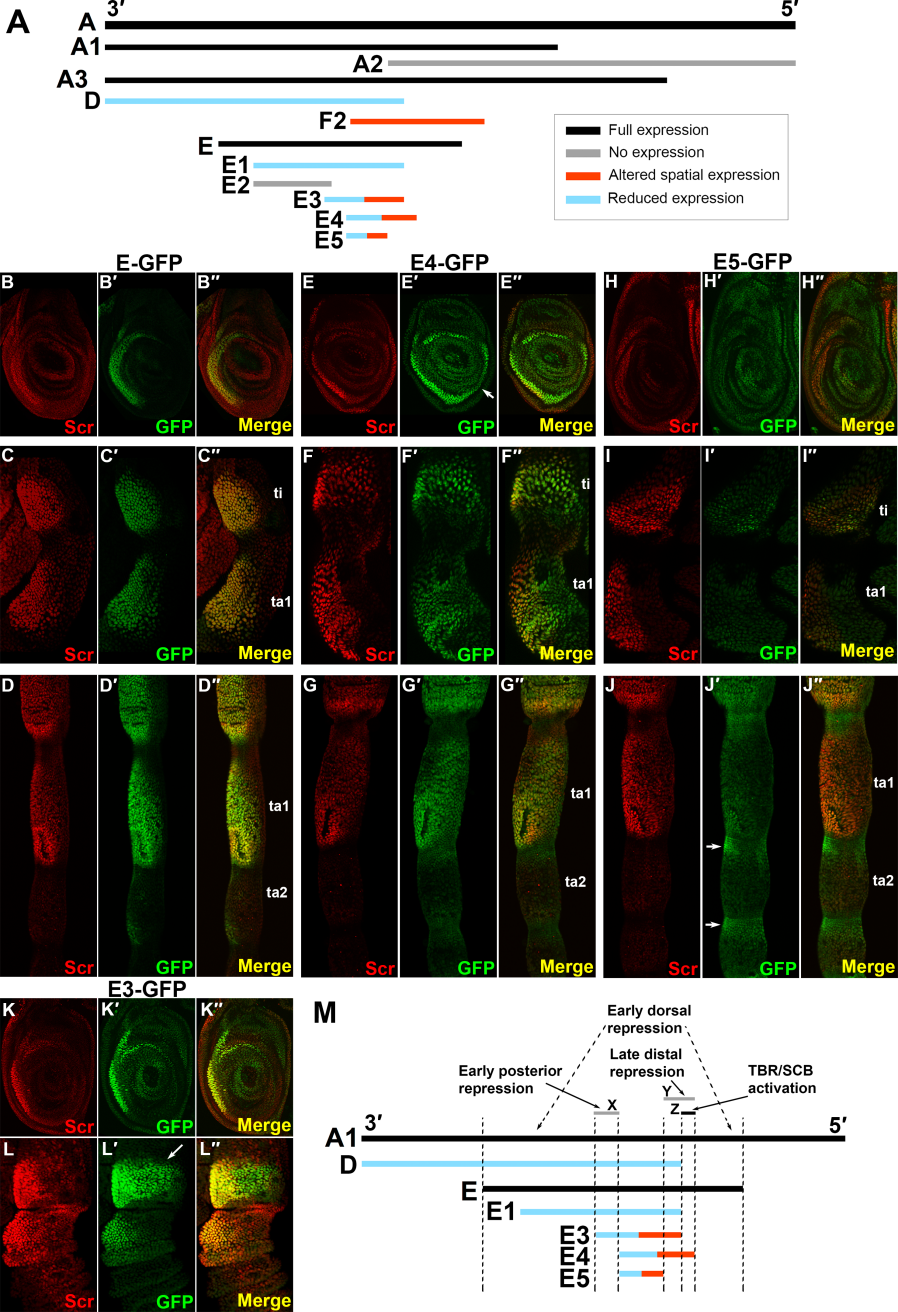
Distinct sequences are required for localized activation and stage-specific repression of *Scr*. A. Sub-clones of fragment A that were tested for enhancer activity (A subset of fragments tested are shown; the complete collection of fragments is shown in S2A Fig). Black bars designate fragments that faithfully reproduce Scr expression in the TBR/SCB primordia; gray fragments direct no expression; red fragments direct altered spatial expression patterns compared to endogenous Scr expression; blue fragments direct lower levels of reporter expression compared to fragment A. Fragments that exhibit both reduced activity and direct altered spatial patterns are designated as both blue and red. B-D". Scr and GFP reporter expression from fragment E in 3rd instar larval leg discs (B-B"), in the first tarsal segment (ta1) and tibia (ti) of 6h APF prepupal legs (C-C") and 24h APF pupal legs (D-D"). In these and all panels, Scr expression (anti-Scr) is shown in red, and reporter expression is shown in green; in images of larval leg discs anterior is to the left and dorsal is up; for prepupal and pupal legs, proximal is up, and antero-ventral aspects are shown. Note that reporter expression recapitulates the elevated Scr expression at all three developmental stages. E-G". Scr and GFP reporter expression from fragment E4 in 3rd instar larval leg discs (E-E"), 6h APF prepupal legs (F-F") and 24h APF pupal legs (G-G"). Note expansion of reporter expression to the posterior compartment (arrow in E') in 3rd instar (E-E") and prepupal legs (F-F"). P/D pattern remains faithful at the pupal stage. H-J". Scr and GFP reporter expression from fragment E5 in 3rd instar larval leg discs (H-H"), 6h APF prepupal legs (I-I") and 24h pupal legs (J-J"). Elevated expression in 3^rd^ instar legs is lost (H-H"). Note low-level tibial expression but lack of elevated expression in ta1 in prepupal legs (I-I"). Also note loss of expression in the SCB primordia of pupal legs in (J-J") and ectopic expression along the P/D axis (arrows). K-K". Scr and GFP expression in a 3^rd^ instar larval leg disc carrying *E3-GFP*. Upregulated reporter gene expression is confined to the anterior compartment, and expression levels are reduced relative that from *ScrE*. L-L". Scr and GFP reporter expression in a prepupal leg (6h APF) carrying *E3-GFP*. Note dorsal expansion of reporter expression relative to the endogenous Scr expression (arrow in L') in the tibia. M. Summary of the upstream enhancer analysis. Regions of *ScrE* necessary for activation or for repression along the P/D axis in pupal legs or along the A/P or D/V axes of 3^rd^ instar and prepupal legs are shown. Region Z is essential for expression around the SCB primordia of male pupal leg and for full expression in the TBR primordia of 3^rd^ instar and prepupal legs. However, additional activating sequences are located in other regions of the enhancer, e.g., the E3 fragment. Sequences mediating dorsal repression map outside of fragment E3 within fragment E. Finally, posterior compartment repression and repression in distal joints of pupal legs are mediated by sequences in regions X and Y, respectively.

With the goal of identifying sequences within fragment A that are necessary for enhancer function, a series of smaller, overlapping sequences from this fragment were tested for enhancer activity in larval, prepupal and pupal legs (Figs 3A and S3A). Of 20 fragments tested, 6 (fragments A2, G, J, F1, F3, E2) did not direct reporter expression in the domain of elevated *Scr* expression at any stage of leg development, while the remaining 14 fragments exhibited full or partial activity (Figs 3 and S3). For the lines bearing reporters with these 14 fragments, we assayed the spatiotemporal pattern directed by each, and quantified GFP signal intensity from a subset of reporter lines (see Methods, S3B Fig, S2 Table). We found that six of these fragments directed expression that faithfully recapitulated that of endogenous *Scr* in the TBR/SCB primordia (fragments A, A1, A3, E, F, K), except for minor variation among individuals in the activity of reporter K at later pupal stages (S4 Fig). Some of the remaining eight fragments showed ectopic dorsal, posterior, or distal expression at different stages of leg development, while others showed reduced expression levels (Figs 3 and S3). In this analysis, fragment E (1.6 kb) (Fig 3A and S3A) was the smallest that fully recapitulated the spatiotemporal pattern of elevated *Scr* expression at all stages of leg development (Fig 3B-D"). Furthermore, fragment E directs comparable, although moderately elevated, expression as compared to fragment A (S5 Fig). The smaller E1 fragment (Fig 3A and S3A) directed proper spatial expression in the TBR primordia, but at a lower level than did the E fragment (36% loss, S2 Table, S3B Fig), and distal/ventral expression associated with the SCB primordia was compromised (S4C-C", S4D Fig). Fragment E was designated *ScrE* and used for further analysis of the upstream enhancer.

Further analyses allowed identification of several regions within the E fragment involved in activation or repression of reporter gene expression (summarized in Fig 3M). Fragment E4, a 439 bp sub-fragment of E (Figs 3A and S3A), is the smallest that can reproduce the sexually dimorphic *Scr* expression pattern in pupal legs; although its pattern is generally faithful, it shows slightly reduced expression in epithelial cells distal/ventral of the sex comb primordia (Fig 3G-G"). E4 also drives expression in the TBR primordia of 3^rd^ instar and prepupal legs, although at reduced levels, particularly in prepupal legs (Fig 3E-F"). E5, a 269 bp sub-fragment of E4, (Figs 3A and S3A), directs minimal expression in the TBR primordia of 3^rd^ instar and prepupal legs (Fig 3H-I"). In addition, elevated reporter expression in the pupal sex comb region was lost; instead, ectopic expression was observed around tarsal joints (Fig 3J-J"). These observations suggest that sequences within the 170 bp at the 5' end of E4 (Fig 3M, region Y) activate elevated reporter gene expression in both the TBR primordia of larval/prepupal legs and around the SCB primordia of male pupal legs. This region is further narrowed to region Z (Fig 3M) by the finding that elevated expression associated with the SCB is strongly reduced in legs carrying *D-GFP* or *E1-GFP* reporter genes (S4C-C" Fig, S3B Fig), while expression in larval and prepupal TBR primordia is reduced (S2 Table) but less affected than that in pupal legs. Although this analysis implicates region Z (Fig 3M) in activation of elevated *Scr* expression during both early and late leg development, additional observations indicate that other *ScrE* sequences can activate expression. For example, fragment A2 (Fig 3A) contains region Z but does not activate elevated expression at any stage, indicating the necessity of sequences 3’ to region Z. In addition, other fragments lacking region Z drive mostly accurate, albeit reduced, expression in the TBR primordia (e.g., fragments E1 and H, Figs 3A and S3A and S3B).

This analysis also defined regions of *ScrE* necessary for repression either along the P/D axis in pupal legs or along the A/P or D/V axes of 3^rd^ instar and prepupal legs. Pronounced ectopic expression is observed near the joints of pupal legs carrying *E5-GFP* (3J-J"), as compared to *E4-GFP* (3G-G"), implicating 5' E4 sequences (region Y, Fig 3M) in distal repression. Elevated Scr and ScrE-GFP expression is confined to the anterior compartment. However, derepression of GFP expression was observed in 3^rd^ instar and prepupal legs bearing an *E4* reporter gene (Fig 3E-F"), while expression is confined to the anterior compartment in *E3-GFP* legs (Fig 3K-K"), suggesting region X (Fig 3M) at the 3’ end of E3 mediates this repression. A similar derepression in the posterior compartment was observed with reporters containing fragments F2 and I, both of which also lack region X (Figs S3B and S6A-A"). On the other hand, elevated expression in the SCB primordia is not expanded. Furthermore, we observe dorsal derepression of E3-GFP (Fig 3L-L") in prepupal legs, implying that sequences mapping outside of E3, but within E, mediate this repression perhaps in response to Decapentaplegic (Dpp), which our genetic studies have implicated in repression (McCallough et al., manuscript in prep). Finally, in the SCB region, we note that expression driven by fragment E1 is stronger and more accurate than expression driven by the larger fragment D, which completely encompasses E1 (Fig 3A and S4C-D"). Comparison of fragments E1, E, H, and D (Figs 3A and S3) suggests the existence of a conditional distal repressor element between the left boundaries of E and D, whose activity is only revealed when region Z (Fig 3M) is deleted.

Combined, these observations suggest that correct spatial and temporal expression of *Scr* requires a delicate balance among multiple activating and repressive elements, mostly located within fragment E, that act in a region- and stage-specific manner to produce the cumulative expression pattern observed for fragment E and the native *Scr* gene (Fig 3M). As described below, further analyses have identified specific sequences that mediate activation or repression of elevated *Scr* expression.

### Conserved domains of the upstream enhancer mediate repression of *Scr* expression along the A/P and P/D axes

**Fig 4:**
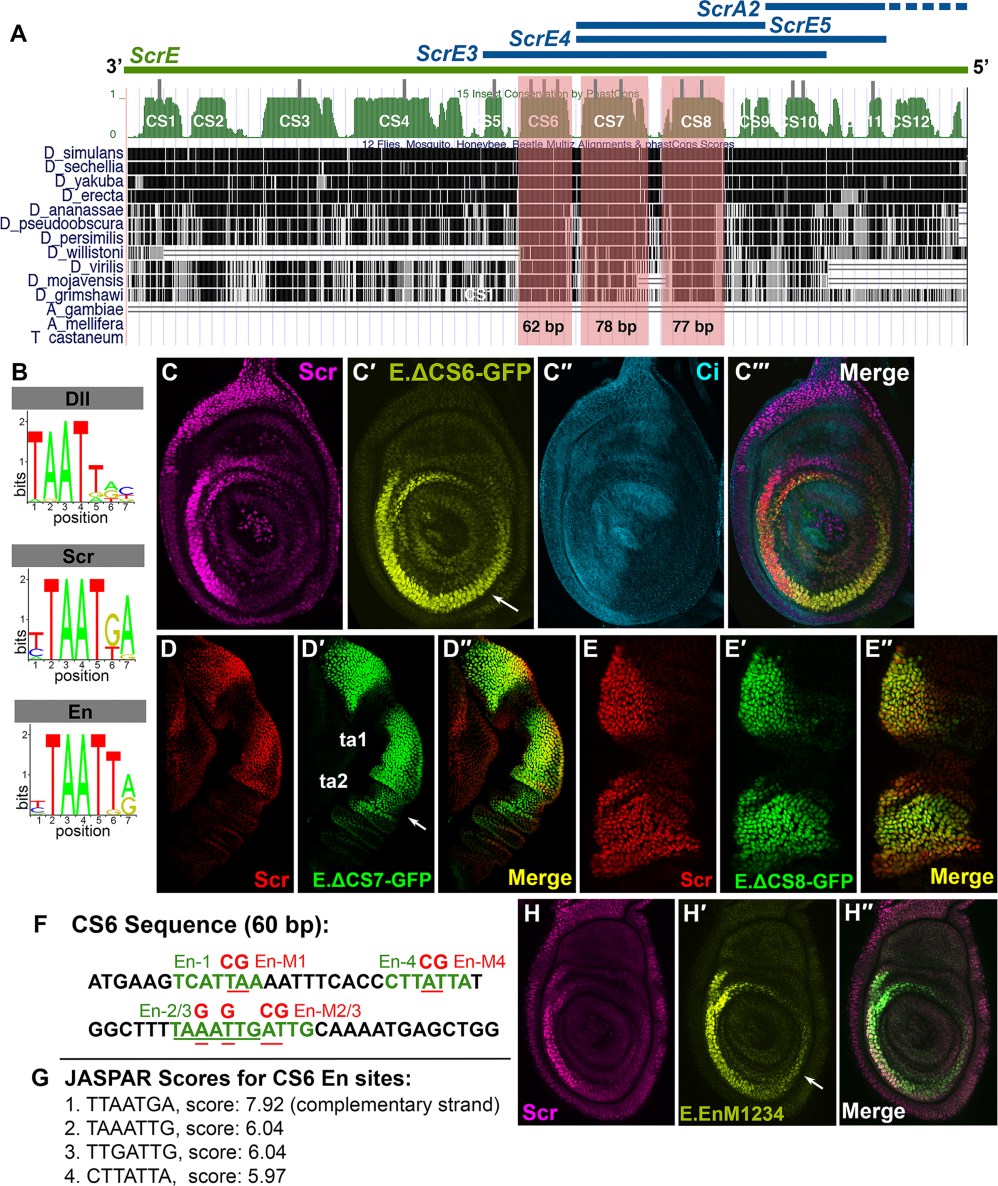
Conserved domains in the upstream enhancer mediate response to leg patterning information along the A/P and P/D axes. A. Map of fragments E, E3, E4, E5 and A2 relative to sequence alignments of 12 *Drosophila* species. Conserved sequence (CS) blocks are numbered 1–12. The gray rectangles indicate high-scoring predicted binding sites for homeodomain proteins, Dll, Scr or En. CS 6, 7 and 8 contain a higher proportion of putative homeodomain binding sites than other CS domains. Each CS block was deleted individually within the context of *ScrE* to generate *ScrE*.*ΔCS6*, *ScrE*.*ΔCS7*, and *ScrE*.*ΔCS8*. B-B". Position weight matrices (PWM) for each TF binding sequences are from the Jaspar Core Insecta database. C-C". Scr and GFP reporter expression in 3rd instar larval leg discs carrying *ScrE*.*ΔCS6-GFP* (C′, green). Ci expression (C″, anti-Ci, cyan) marks the anterior compartment of the 3rd instar leg disc. Note posterior compartment expansion of reporter expression (arrow in C') as compared to Ci and endogenous Scr expression (C, anti-Scr, magenta). Reporter gene expression in these legs exhibits slightly decreased GFP intensity as compared to ScrE-GFP (S3B Fig, S2 Table). D-D". Scr (red) and GFP reporter (green) expression in a prepupal leg (6h APF) carrying *ScrE*.*ΔCS7-GFP*. Note the distal expansion of reporter expression (arrows). E-E". Scr (red) and GFP reporter (green) expression in a prepupal leg (6h APF) carrying *ScrE*.*ΔCS8-GFP*. Reporter gene expression in these legs exhibit decreased GFP intensity as compared to ScrE-GFP (S3B Fig, S2 Table). F. The wild type 60 bp CS6 sequence is shown with putative En binding sites (En1-4) in green. Sites 2 and 3 partially overlap, and site 2 is underlined in green. All four putative En binding sites were mutated; nucleotides that were mutated are underlined in red, and changes are shown in red above the wildtype sequence, (En.M1-4). G. JASPAR scores for the four putative En binding sites. The first site shown on the left is located in the complementary strand. H-H". A 3rd instar leg disc showing reporter expression from a transgenic line carrying *ScrE-GFP* with mutations in the En-1, En-2/3 and En-4 sites (H′, green). Reporter expression is expanded to the posterior compartment as compared to endogenous Scr expression (H, magenta).

As described above, we have identified regions of the *ScrE* enhancer that are essential for activation and repression along the A/P, D/V and P/D axes. To further define sequences within the *ScrE* enhancer that respond to patterning inputs during leg development, we aligned the *ScrE* fragment from 12 Drosophila species and found multiple conserved sequences (CS1-12, Fig 4A), of which CS6-8 exhibit the highest degree of conservation (Fig 4A). CS6-8 are contained within the *E3* fragment (Fig 4A), which activates reporter gene expression within the *Scr* expression domain, although at a reduced level (Figs 3A, K-L" and S3, S2 Table).

To determine if CS6-8 are essential for proper *ScrE* directed expression, each block was individually deleted from the E fragment, and corresponding reporter transgenic lines were generated, which were named ScrE.ΔCS6-GFP, ScrE.ΔCS7-GFP and ScrE.ΔCS8-GFP (Fig 4). Expression directed by each deletion line was assayed in larval, prepupal and pupal legs. CS6 is contained within region X in fragment E3 (Fig 3M), which, as shown above, we found is required for posterior compartment repression (Fig 3E-E",M). Deletion of CS6 resulted in ectopic reporter expression in the posterior compartment of third instar leg discs and prepupal legs (Fig 4C-C"), suggesting that CS6 contains sequences that mediate response to a posterior compartment-specific repressor, possibly En.

Legs bearing ScrE.ΔCS7-GFP exhibited distal expansion of reporter expression to the ta2 segment (Fig 4D-D"), implying loss of response to repression in the distal leg, potentially by Bric-a-brac1 and Bric-a-brac 2 (Bab1 and Bab2), which have been shown to inhibit distal *Scr* expression [35] and will be referred to collectively as Bab. Lastly, deletion of CS8 did not result in an altered spatial pattern (Fig 4E). However, when GFP intensity levels from *ScrE-GFP* and *ScrE*.*ΔCS8-GFP* reporter lines were compared, reduced levels of expression were observed from the latter (S3B Fig, S2 Table).

### Conserved HD consensus binding sites mediate posterior compartment repression of *Scr* expression

Our observations identify specific sequences within *ScrE* that are necessary for correct spatial modulation of *Scr* expression levels. Genetic studies, expression analysis, and this study suggest two candidate homeodomain (HD) transcription factors that might act through *ScrE*, En and Dll (McCallough, in prep). Therefore, we searched the *ScrE* sequence for putative HD binding sequences. HD transcription factors are known to bind similar short AT-rich sequences, making it cumbersome to identify specific response sites for different HD proteins. However, sequence-specific binding preferences have been reported for various classes of HD transcription factors [36, 37]. To refine our search for putative HD binding sites that might mediate response to Dll and En, we used the JASPAR database for Core Insecta to search CS1-12 of *ScrE* for preferred binding sequences for Dll and En (Fig 4B). We also included putative Scr binding sites, as it has been reported that Hox genes can auto-regulate their expression in various contexts [38–41].

We identified multiple putative HD binding sites and noted that half of these sites were concentrated in the CS6-8 blocks (Fig 4A and 4B). Given the observation that *Scr* reporter genes lacking CS6 were expressed in the posterior compartment, we hypothesized that CS6 contains En response sequences. Four putative HD binding sites were identified within CS6, two of which overlapped (sites En-1, the overlapping En-2/3 and En-4 in Fig 4F; JASPAR scores are shown in 4G). All four sites were mutated in *ScrE* (*ScrE-En*.*M1234*), which resulted in derepression of reporter expression in the posterior compartment, similar to deletion of the entire CS6 (Fig 4F, H-H"). Separate mutations in En-1 or En2/3 did not result in derepression of reporter expression in the posterior compartment (S6B-C" Fig). These observations suggest that En repression is mediated via a combination of En sites within CS6. However, reporter expression directed by ScrE.ΔCS6-GFP is lower in the posterior than in the anterior compartment (S2 Table), suggesting the existence of additional En or other repressor response sequences in the E fragment.

In summary, we observe that distinct sequences in *ScrE* mediate response to patterning inputs along the three different axes. Specifically, we have identified sequences required for repression in the posterior compartment and in regions dorsal and distal to the normal domain of *ScrE* expression. Repression in the posterior compartment is mediated by HD binding sites, and below we show that several other putative HD binding sites are required for full enhancer activity.

### Distalless activates *Scr* expression through the upstream enhancer, and conserved HD consensus sites are essential for enhancer activity

*Dll* and elevated Scr expression partially overlap in third instar larval discs and prepupal legs (McCallough et al., manuscript in prep). *Dll* expression extends from mid-tibia to ta5, whereas elevated *Scr* expression ends at the distal end of ta1. As mentioned previously, genetic studies implicate Bab in defining the distal boundary of *Scr* expression [35]. Based on the expression data and genetic evidence, we hypothesized that *Dll* activates *Scr* directly through the *ScrE*.

**Fig 5:**
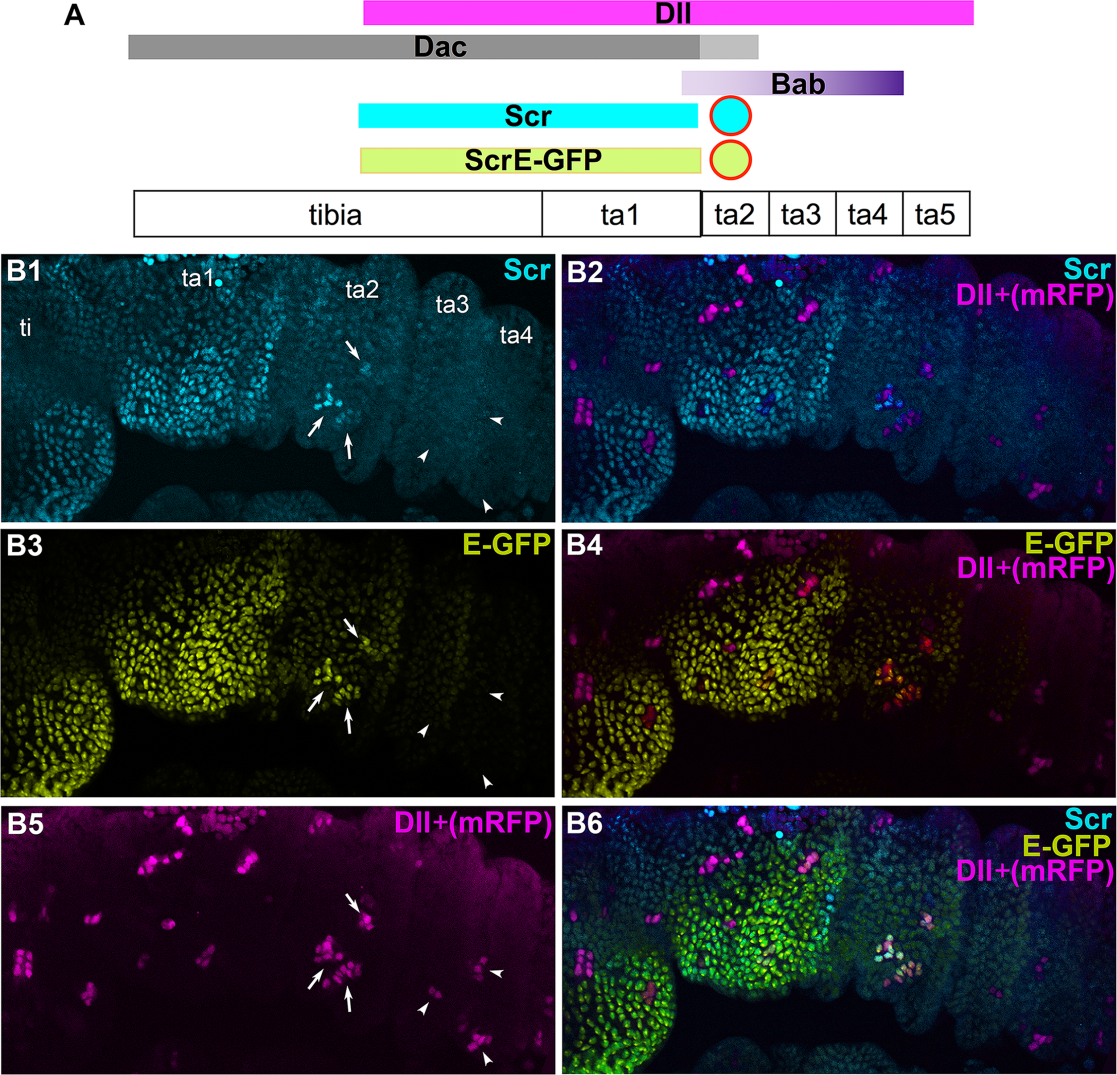
Dll acts through the upstream enhancer to activate *Scr* expression. A. Diagram showing the positions of gain-of-function *Dll* clones along the P/D axis of the leg. The expression domains of P/D patterning genes *Dll* (magenta), *dac* (gray) and *bab* (purple) are shown relative to elevated Scr expression (cyan) and to Scr reporter expression (green). The circles above ta2 represent gain-of-function *Dll* clones that activate endogenous Scr expression as well as reporter expression. B1-B'6. *Dll* gain-of-function clones in a 6h APF prepupal leg are marked with mRFP expression (magenta). ScrE-GFP expression is shown in green (B3,B4,B6); endogenous Scr expression is shown in cyan (anti-Scr; B1,B2, B6), and mRFP expression, marking *Dll* gain-of-function clones is shown in magenta (B2,B4-B6). Arrows designate gain-of-function *Dll* clones located in ta2 that activate Scr expression, as well as expression from the upstream *Scr* enhancer, *ScrE*. Arrowheads indicate *Dll* gain-of-function in distal segments, ta3 and ta4 in which neither endogenous Scr nor Scr-GFP expression is activated.

To test whether *ScrE* responds to Dll, we generated *Dll* gain-of-function clones and observed cell-autonomous ectopic reporter expression from clones located in the ta2 segment (Fig 5). Surprisingly, *Scr* expression was not activated in clones located in ta3 or ta4 (Fig 5), which could be due to competition with a repressor of *Scr* expression, possibly Bab. Bab inhibits elevated *Scr* expression in segments ta2 and more distal tarsal segments [35] and is expressed in a distal to proximal gradient [42], suggesting that Dll might overcome Bab mediated repression in ta2 but not in more distal segments, where Bab levels are higher. Together, these findings suggest that Dll acts through *ScrE* to activate *Scr* expression.

**Fig 6:**
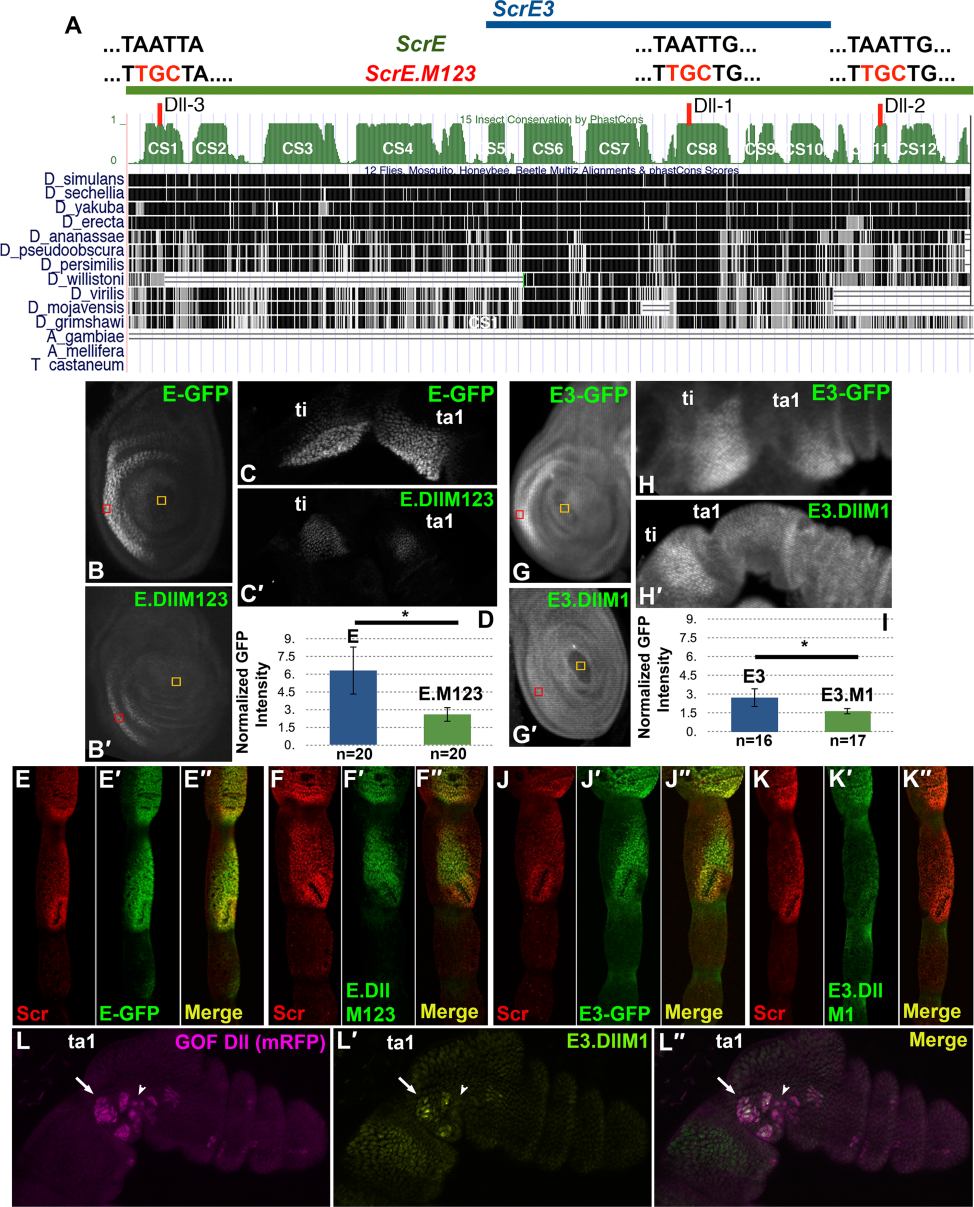
HD consensus binding sites are required for elevated levels of reporter expression throughout leg development. A. Map of fragments E and E3 relative to sequence alignments of 12 *Drosophila* species showing three highly-conserved consensus Dll sites in black above the map of E. Red indicates nucleotide substitutions in mutated sequences. Note that *E3* contains only one of the three Dll sites (Dll-1). Conserved sequence (CS) blocks are numbered 1–12. B-C'. Z-projections of third instar leg discs (B-B') and prepupal legs (C-C') from transgenic lines carrying *ScrE-GFP* or *ScrE*.*DllM123-GFP*. Note that the reporter expression is lower in transgenic animals that carry the *ScrE*.*DllM123* (B',C') construct versus *ScrE* (B,C). Red and yellow boxes indicated regions used for quantifications (see panel D). D. GFP signal quantification of identically imaged 3rd instar legs carrying either the *ScrE-GFP* or *ScrE*.*DllM123-GFP* reporter (see Methods). GFP intensity was measured from a square region in the middle of the crescent-like region of elevated expression in Z-projections of third-instar leg discs and normalized to an identically sized area in the center of the disc (red and yellow squares in panels B,B' and G,G'). Normalized GFP intensity values were averaged from multiple animals (n, in panels D and I); standard deviations were calculated, and a two-tail t-test was used to determine p-values. The signal intensity of ScrE.DllM123-GFP expressing legs is significantly lower than that of ScrE-GFP expressing legs (*p = 1.3X10^-5^). Quantifications data and statistics for larval and prepupal legs in this figure are shown in S2 Table. E-E". Scr (red in E,E") ScrE-GFP (green in E',E") expression in male pupal legs. F-F". Scr (red in F,F") and ScrE.DllM123-GFP (green in F',F"). GFP expression is lower in the region around SCB (F',F") compared to Scr protein (F) and ScrE-GFP (E') expression. G-H'. Z-projections of third instar leg discs (G-G') and prepupal (H-H') from two transgenic lines, *E3-GFP* and *E3*.*DllM1-GFP*. Note that reporter expression is lower in transgenic animals that carry the *E3*.*DllM1-GFP* (G',H') construct as compared to E3-GFP expression (G,H), and the reporter expression in ta1 of prepupal legs is not above baseline levels (H'). I. GFP signal quantification of identically imaged 3rd instar legs carrying either the *E3-GFP* (G) or *E3*.*DllM1-GFP* (G') reporter (see Methods). Regions of leg discs used for measurements of GFP intensity and statistics are shown in G and G' and are as described above in the legend for panel D. The signal intensity of E3.DllM1-GFP expressing legs is significantly lower than that of ScrE.DllE3-GFP expressing legs (*p = 1.3X10^-5^). Note that the GFP intensity in ScrE.DllE3-GFP expressing legs is already low relative to ScrE-GFP (D and S2 Table). J-J". Scr (red in J,J") and E3-GFP (green in J',J") expression in male pupal legs. Note reduced E3-GFP (J') expression in the region around SCB, as compared to E-GFP (E'). K-K". Scr (red in K,K") and E3.DllM1-GFP (green in K',K") expression in male pupal legs. Note that E3.DllM1-GFP (K') expression in the region around SCB is reduced as compared to E3-GFP (J'). L-L". *Dll* gain-of-function clones in a 6h APF prepupal leg are marked with mRFP expression (magenta in L,L"). E3.DllM1-GFP expression (green in L',L") within the TBR primordia is partially rescued in subset of clones (e.g., clone designated by arrow), but is not rescued in other clones (e.g., clone designated by arrowhead).

To determine whether Dll might act directly through *ScrE*, we searched for putative HD binding sites in *ScrE* that match the reported preferred Dll-binding sites [37]. Three putative Dll sites were identified; two sites, Dll-1 in CS8 and Dll-2 in CS11, have the same sequence, TAATTG, and the third site, Dll-3 in CS1, has the sequence TAATTA (Fig 6A). To test whether these putative Dll sites are necessary for *ScrE* reporter expression, we mutated the TAAT core sequence in each Dll site (*ScrE*.*DllM123*) (Fig 6A). Quantification of reporter expression (see Methods) directed by *ScrE* and *ScrE*.*DllM123* in larval and prepupal legs (Figs 6B–6D and S7, S2 Table) showed that in repeated GFP intensity measurements in the region of elevated expression, ScrE.DllM123-GFP expression is consistently lower compared to that of ScrE-GFP. This indicates that reporter gene expression was compromised by mutation of the three putative Dll sites. Moreover, ScrE.DllM123-GFP expression is also diminished in the presumptive SCB primordia relative to ScrE-GFP (Fig 6E-F"). These findings are consistent with the hypothesis that Dll acts directly through *ScrE* to activate *Scr* expression, and that these sites are necessary throughout leg development. However, because the ScrE.DllM123-GFP expression is not completely abrogated, it is likely that additional sequences mediate activation.

Mutation of three putative Dll binding sites in *ScrE* results in substantially reduced expression in the TBR/SCB primordia, but expression is not eliminated (Figs 6B–6F"). Therefore, we shifted our focus to the E3 fragment, which has just the Dll-1 site and directs expression that is 47% of that directed by *ScrE-GFP* (S7 Fig, S2 Table). To further test Dll activation of elevated *Scr* expression, we generated a transgenic line that is mutant for Dll-1 in the context of the E3 fragment (*E3*.*DllM1*) (Fig 6A). In third instar leg discs carrying the *E3*.*DllM1-GFP* reporter, expression in the TBR primordia was only 1.5-fold above baseline (see Methods and Fig 6D legend), whereas it was elevated nearly 3-fold in leg discs bearing the non-mutant *E3-GFP* reporter (Fig 6G-G' and 6I). Similarly, in prepupal legs, the reporter expression levels in ta1 were no higher than in more distal tarsal segments, suggesting that elevated expression in the ta1 segment was eliminated (Fig 6H-H' and S2 Table). At the pupal stage, elevated expression in the TBR/SCB region of ta1 was substantially reduced in legs carrying *E3*.*DllM1-GFP* as compared to *E3-GFP* legs (Fig 6J-K"). On the other hand, some elevated expression in the distal tibia was still observed at both prepupal and pupal stages (Fig 6H-H',6K-K").

*Scr* reporter genes direct expression in legs from all thoracic segments (S1C-C" Fig), but not in the wing disc. Therefore, we were surprised to observe that clones over-expressing Dll can induce ScrE-GFP, but not endogenous Scr expression, in the wing disc (S8A-A‴ Fig). This finding provides further support for the hypothesis that Dll activates *Scr* expression through *ScrE*. Our observations also indicate that regulation of *ScrE* by En in the wing mimics that in the leg, as reporter gene expression is detected only in anterior compartment clones (S8A-A‴ Fig).

Our findings indicate that conserved HD consensus sites are necessary for full activity of the *ScrE* enhancer. Further, the observation that *ScrE* is responsive to Dll suggests that Dll might act through these sites as an activator of elevated *Scr* expression. We asked whether these sites are necessary for full response of *ScrE* to Dll by assaying expression of E3.DllM1-GFP in *Dll* gain-of-function clones. As described above, E3.DllM1-GFP expression in ta1 is severely compromised and is barely elevated above the low-level expression in cells outside the TBR primordia, while in the tibia, expression is somewhat higher but still reduced. We observed that *Dll* gain-of-function clones in ta1 partially rescued E3.DllM1-GFP expression (Fig 6L-L"). A subset of *Dll* gain-of-function clones exhibited expression that was not substantially different from surrounding cells, while other clones showed higher but variable expression (Fig 6L-L"). We also assayed response of the *ScrE*.*DllM123* reporter in *Dll* gain-of-function clones in wing discs and observed that ScrE-GFP expression was activated in 93.5% (43/46) of clones in the wing as compared to ScrE.DllM123-GFP, which was expressed in 44% (32/73) of clones (S8B–S8C Fig).

The incomplete elimination of elevated expression driven by *E3*.*DllM1* and *ScrE*.*DllM123* could be due to the presence of additional Dll response sites within these fragments, which is supported by the finding that *E3*.*DllM1* is still partially responsive to Dll. It is also plausible that Dll can act through less optimal HD sites in *ScrE*.*DllM1* or *ScrE*.*DllM123*, such as En sites in CS6, but only when it is present at high concentrations. On the other hand, we have not ruled out the possibility that an HD transcription factor other than Dll acts through the putative Dll1-3 sites to activate *Scr* expression and that Dll acts indirectly to regulate *SrcE* directed expression. Together, our observations demonstrate that *ScrE* is responsive to Dll and that HD consensus sites are essential for full activity of the *ScrE* enhancer.

## Discussion

### The upstream *Scr* enhancer directs sexually dimorphic expression and is required for TBR and sex comb development

In this study, we examined two enhancers of the Hox gene *Scr*, both of which drive expression in the T1 leg. We followed expression directed by the two enhancers from early (larval) through late (pupal) stages of T1 leg development and found that, while both direct expression in the TBR primordia, the upstream enhancer more faithfully reproduces the precise spatial boundaries of elevated *Scr* expression over time, and specifically functions to direct elevated expression in the SCB primordia. Importantly, deletion of the upstream enhancer results in loss of TBRs in both male and female legs and SCBs in the male T1 leg, demonstrating that this enhancer is necessary for the development of segment- and sex-specific leg sensory organs and that the intronic enhancer function is not sufficient to promote development of either TBRs or SCBs. Furthermore, the intronic enhancer is not necessary for development of either TBRs or SCBs, a surprising finding given the activity of this enhancer in the TBR primordia during larval and prepupal development. The existence of two enhancers that activate expression in the TBR primordia evokes prior reports on genes that possess multiple “shadow” or “distributed” enhancers, separable *cis*-regulatory elements that direct similar patterns of expression [43, 44]. Several studies have shown that redundant shadow enhancers allow robust and reproducible gene expression under conditions of stress, leading to the proposal that such enhancers may mediate canalization [45, 46]. Although it appears that the intronic enhancer is not essential for development of TBRs and SCBs, this does not rule out a function for this enhancer under conditions of environmental stress or in variable genotypic backgrounds.

### Modular repression elements and diffuse activation sites establish localized elevated *Scr* expression in T1 legs

Our investigations have provided insight into the mechanisms involved in establishing elevated levels of *Scr* expression at various stages of leg development. In addition to the two enhancers that drive elevated expression in the TBR and SCB primordia, we find that promoter-proximal sequences direct low-level expression in the rest of the leg and appear to be necessary for full activity of at least the intronic enhancer. In the upstream enhancer, activating sequences necessary for maximum expression are spread over a fairly wide region, while distinct repressive sequences are necessary for faithful spatial expression at different developmental stages. Separate regions within the *ScrE* fragment mediate repression during larval and prepupal stages in the posterior compartment, distally in the ta2 segment, and in the dorsal leg. Finally, some sequences are specifically required for correct expression in the SCB primordia at the pupal stage.

While endogenous *Scr* expression in thoracic discs is almost exclusively restricted to T1 legs [3, 4], we observe that both the intronic and upstream enhancers direct expression in T2 and T3 legs in patterns that are similar to that in T1 legs. There are two plausible explanations for this observation. First, these enhancers may have functions in the development of T2 and T3 legs, but the lack of native Scr expression in T2 or T3 legs [3, 4] argues against this hypothesis. In principle, the intronic and/or upstream enhancers could also regulate *Antp*, which is located close to the upstream enhancer (Fig 1). However, previous studies have shown that although *Antp* is expressed in all three pairs of leg discs, by third larval instar, its expression is largely restricted to proximal cells that will give rise to thoracic structures, with only weak expression in leg tissue of late T2 leg discs [47, 48]. Finally, we note that the deletion of either the upstream or the intronic enhancer has no observable phenotype in the T2 and T3 legs (S2A and S2B Fig).

The more likely explanation is that the expression of intronic and upstream enhancers in T2 and T3 legs is due to the integration of reporter constructs at heterologous sites that lack the silencing elements that are necessary for segment-specific *Scr* expression. This hypothesis would also explain our observation that *Dll* gain-of-function clones could activate reporter gene but not endogenous *Scr* expression in the wing disc, which is derived from the T2 segment. The notion that activity of the intronic and upstream enhancers in T2 and T3 legs is due to the absence of sequences that mediate silencing is supported by prior investigations. For instance, mutations in Polycomb group genes, which are known to maintain repressed gene states [49, 50], are associated with ectopic *Scr* expression in T2 and T3 legs and formation of ectopic sex combs on these legs [4, 31]. Moreover, several reports suggest that specific sequences within the 10kb XbaI fragment, which contains the upstream enhancer, function in silencing of *Scr* expression [4, 29, 30, 32] and are bound by the Enhancer of trithorax and Polycomb (ETP) proteins, DSP1 and Corto [28, 32]. The regions bound by DSP1 and Corto do not overlap *ScrE* and only partially overlap fragment A at its 5’ end (S5 Fig) [28, 32]. DSP1 is necessary for maintaining expression in T1 legs, while Corto, which is known to interact with complexes of Polycomb group proteins, is involved in maintaining repression of *Scr* in T2 and T3 legs [32]. Our observations, combined with those of other investigators, are consistent with the suggestion that the *Scr* leg enhancers have the potential to be active in all legs but are normally maintained in a repressed state in T2 and T3 legs by silencing elements associated with the *Scr* locus.

### HD consensus binding sites mediate both posterior compartment repression and activation of elevated *Scr* expression

Our results show that Dll, a transcription factor with conserved functions in limb development [51], acts through the upstream enhancer to activate elevated *Scr* expression in T1 legs. These conclusions are supported by the observation that Scr and ScrE-GFP expression is activated in *Dll* gain-of-function clones, although the response is spatially limited, which we attribute to repressive inputs along the three axes, from repressors such as En in the posterior compartment and Bab in the distal leg. Further support is provided by the surprising observation that reporter gene expression is activated in *Dll* gain-of-function clones throughout the anterior wing pouch. Mutation of HD consensus sites predicted to be preferentially bound by Dll resulted in reduced reporter gene expression, but mutant enhancers were still partially responsive to over-expression of Dll. This could be due to the existence of additional Dll binding sites in the mutant enhancer or to Dll acting through sub-optimal HD binding sites, perhaps En- sites, under conditions of over-expression. However, it is also possible that Dll function in regulation of *Scr* is indirect, and an unknown HD factor acts through the identified sites to activate *Scr* expression in the TBR/SCB primordia.

HD consensus binding sites binding sites within the conserved CS6 also mediate posterior compartment repression, likely in response to the En repressor. This suggestion is supported by the finding that Dll activation of *Scr* obeys the A/P boundary in both wing and leg discs, both of which express En in the posterior compartment. While we cannot rule out the possibility that Dll can act through En sites or vice versa to regulate *ScrE* directed expression, it appears that separate HD binding sites mediate activation and repression because mutation of putative Dll binding sites resulted in loss but not expanded expression, and deletion of CS6 caused expanded but not substantially reduced expression.

### Modulated Hox gene expression in leg imaginal discs contributes to generation of cell type diversity

Varying levels of Hox gene expression within developing Drosophila limbs have been observed in multiple contexts [1–8], but the relevance and regulation of this heterogeneous Hox gene expression have not been extensively investigated. We have previously found that elevated expression of *Scr* in specific regions of prepupal legs functions to alter the T1 leg proneural prepattern [10], while sex-specific elevation of *Scr* expression in the SCB primordia is required for differentiation of sex comb vs transverse bristle row morphologies [9]. Here we have identified an enhancer that is necessary for upregulated *Scr* expression in both the TBR and SCB primordia and have determined that this enhancer exploits the regulatory network common to all Drosophila legs to pattern the T1 leg-specific sense organs.

It is noteworthy that in larval and pupal legs, *Scr* expression is modulated by the T1 intrasegmental patterning network. This represents a reversal of regulatory interactions observed in the embryo, in which Hox genes modulate segmentally repeated expression patterns of multiple transcription factors and signaling molecules. For example, *Ubx* and *abdominal-A* inhibit leg formation in abdominal segments via repression of *Dll* expression [52]. By illuminating the position of *Scr* in the leg regulatory network at later stages, our work advances our understanding of the regulatory mechanisms that give rise to specific morphological features of adult limbs. Cross-species analyses of the *cis*- and *trans*-regulatory elements identified in this study are likely to reveal mechanisms involved in the evolution of morphological novelties, such as the diverse sex combs of different *Drosophila* species.

The formation of distinct features of insect limbs in response to differential Hox gene expression has been observed in insect groups outside Drosophila, implicating this as an important mechanism of morphological diversification. Expression of *Scr* in a dorsal tibial patch of Oncopeltus T1 legs, for example, marks a tibial comb, and knockdown of *Scr* function results in altered morphology of the bristles that make up this comb [53–55]. Similarly, *Scr* expression is correlated with formation of grasping structures on the T1 legs of praying mantis (Tenodera) [54]. Modulated expression of *Ubx* in the Drosophila T2 or T3 leg primordia is associated with patterning of specific features, such as suppression of trichome development on the T2 leg femur [6] and formation of TBRs in the posterior compartment of T3 legs [10]. Interestingly, spatially defined expression of *Ubx* in T3 legs of the house cricket (*Acheta domestica*) underlies the enlargement of specific regions of these legs [56]. Combined, these investigations indicate that further study of Hox gene regulation in response to intrasegmental patterning networks will provide new insights into the mechanisms underlying the formation of diverse phenotypic features.

## Methods

### Fly stocks, culture and genetic manipulations

*Drosophila* lines were grown on standard yeast extract-sucrose medium. The following lines were used for site-specific insertion of GFP and Gal4 reporter lines: y[1] v[1] P{y[+t7.7] = nos-phiC31\int.NLS}X; P{y[+t7.7] = CaryP}attP40 [57], y[1] sc[1] v[1] P{y[+t7.7] = nos-phiC31\int.NLS}X; P{y[+t7.7] = CaryP}attP2 [57]. The following strains were used for genetic studies: w[1118] P{w[+mC] = UAS-GFP.nls}14), w[1118]; [58], P{ry[+t7.2] = PZ}Dll[01092] cn[1]/CyO; ry[506] [59], OregonR and y w hsFLP;UAS-*Dll*/CyO (gift from Grace Boekhoff-Falk) [60]. w; *Scr*^*ΔCRE(upA-B)*^/TM3 and w; *Scr*^*ΔCRE(upA-B)*, w+^/TM6 were used to study the phenotypic effect of Scr upstream CRM deletion.

Flip-out clones over-expressing *Dll*, marked with RFP, were induced in y w *hs*-FLP; UAS-*Dll*/*Scr*.*Enhancer*-GFP; GAL4-Act5C(FRT.CD2), UAS-RFP/+ third-instar larvae (48-96h AEL). Larvae were heat shocked at 37°C for 10 minutes and dissected 44 to 96h later.

### Immunofluorescence and microscopy

Third-instar larval imaginal discs were prepared and stained using standard procedures. White prepupae from heat shocked vials were aged to and dissected at 6 hours after puparium formation (APF). Prepupal legs were treated as previously described [61]. Primary antibodies used include: mouse α-Scr [1:25] [3], mouse α-Ubx [62], rat α-Ci [1:1] (gift from R. Holmgren) [63].

To obtain precisely synchronized cohorts of pupae, white prepupae were collected from culture bottles, sexed, placed on a moist Kimwipe in a Petri dish, and aged at 25°C in 70% humidity. Once aged, the pupae were attached to sticky tape and cut with a razor blade into ventral and dorsal halves. The ventral halves were removed from the pupal case and fixed in 4% paraformaldehyde (EM grade, Polysciences) in 1XPBS for 0.5–1 h at room temperature. Samples were washed twice for 10 min each in TNT (0.1M Tris-HCl, 0.3M NaCl, 0.5% Triton X-100, pH 7.4) and transferred to a depression glass. Pupal cuticle was ruptured at the base of each leg using forceps, and the leg was severed near the distal femur/proximal tibia boundary and pulled out of its pocket of pupal cuticle. Dissected legs were blocked for 30 min at room temperature in image iT FX signal enhancer (Thermo Fisher Scientific). Legs were incubated with the primary antibody overnight at 4°C. Samples were then washed several times in the TNT and incubated overnight at 4°C with secondary antibodies. Stained samples were washed three times in TNT buffer and mounted in Fluoromount 50 (SouthernBiotech). The primary antibodies used were mouse anti-Scr 6H4.1, 1∶10 (Glicksman and Brower 1988). The secondary antibodies were AlexaFluor 594, 1∶200 (Invitrogen, Carlsbad, CA).

Fluorescent images were collected as apotomized Z-stacks for larval discs and prepupal legs on a Zeiss Axiovert 200M or Zeiss Apotome.2 with Axiocam 506. Pupal images were collected on Olympus FluoView 1000 confocal microscope. Images were processed after collection using either Image J software or Adobe Photoshop, and all adjustments were uniformly applied to entire images.

For visualization of microchaete pattern in adult legs, animals were dehydrated through an ethanol series (70%, 80%, 90%, 95%, 100%) and mounted in GMM [64]. Bright field image acquisition was used to capture adult leg images.

### Reporter constructs and site-directed mutagenesis

Fragments A, B and C were initially cloned into pStinger GFP vector [65]. The Scr promoter was cloned into pStinger using BamHI and KpnI sites and the enhancer fragments were cloned upstream of the promoter sequence using KpnI and NheI sites. Subsequent fragments were cloned into the pS3aG vector that allows site-specific integration, as described below. pS3aG was a gift from Thomas Williams (Addgene plasmid # 31171) and it was modified to generate pS3aG-ScrP by replacement of the *hsp70* promoter with the *Scr* promoter via restriction sites XhoI and NheI.

Genomic DNA fragments from the *D*. *melanogaster Scr* locus were amplified via standard PCR (Platinum Pfx, Invitrogen or OneTaq, NEB) from BAC clone RP98-32J3 (BAC PAC Resources) using primer pairs with KpnI and NheI or AscI restriction sites. Refer to S3 Table for the complete list of primers used in this study. All fragments were initially cloned in the TOPO plasmid (pCR-Blunt Topo-II or pCRII Vector, Invitrogen) and then transferred to the pS3aG-ScrP vector. TOPO and pS3aG-ScrP plasmids were digested with KpnI and NheI or AscI enzymes (NEB), and were gel excised and purified (Qiagen) before ligation (T4 DNA ligase, Invitrogen and Promega).

All site mutations were generated using the QuikChange Site-Directed Mutagenesis Kit (Stratagene). CS block deletions were performed via overlap extension PCR [66]. pBPGAL4.2Uw-2 plasmid was a gift from Gerald Rubin (Addgene plasmid # 26227) [67]. The Gateway cloning system was utilized to clone the upstream enhancer fragment E into pBPGAL4.2Uw-2 (pBPGAL4.2Uw-2-ScrE) using the same attP40 insertion site on the second chromosome for generation of the ScrE-DSCP-Gal4 strain. The Drosophila synthetic core promoter (DSCP) sequence in pBPGAL4.2Uw-2-ScrE was replaced with the *Scr* promoter using FseI and KpnI restriction sites for generation of the ScrE-ScrP-Gal4 line.

All constructs were sequence-verified. PhiC31-mediated germline transformation was performed as described [57]. Transgenic strains were generated by Rainbow transgenic lines, Inc., by BestGene Inc., or in the Kopp lab.

### Generation of the CRISPR line

To generate the pGX-2attp_WN_Scr_A-B replacement donor [68], 5.299kb 5'-homology arm and 5.127kb 3'-homology arm were amplified and initially cloned in the pCR4-TOPO Vector (Invitrogen) and pCRII Vector (Invitrogen) respectively. The 5'-homology arm was amplified (OneTaq, NEB) with a forward primer containing a NotI site and a reverse primer containing a NheI site. The 3'-homology arm was amplified (OneTaq, NEB) with a forward primer containing an AscI site and a reverse primer containing a StuI site. Refer to S3 Table for the complete list of primers used in this study. The 3'-homology arm was ligated into AscI and StuI sites of pGX-2attp_WN vector [68]. Subsequently the 5'-homology arm was ligated into pGX-2attp_WN vector containing the 3'-homology arm. sgRNA were designed using http://tools.flycrispr.molbio.wisc.edu/targetFinder/ as described [69] and cloned into the *U6b*-sgRNA-short vector as previously described [70]. sgRNA primer sequences are listed in S3 Table. Transgenic flies were generated by The Best Gene, Inc., using y[1] M{vasCas9.RFP}ZH-2A w[1118] strain (BDSC#55821) as a donor for germline transformation and identified using the w[+] marker present in the donor construct. Verification of founder knock-out lines that contain the w[+] marker and position of w[+] marker in the genome was done by PCR and sequencing.

### Quantification of GFP intensity

For each reporter construct, average of GFP intensity was obtained from >15 pro-thoracic third instar larval discs or prepupal legs (6h APF). All transgenic lines were grown and fixed under the same conditions. Z-projections were analyzed with ImageJ software. GFP intensity was measured from a square region in the middle of the crescent-like Scr expression pattern in third-instar leg discs and normalized to an identically sized area in the center of the disc (Fig 6 B-E). The normalized GFP intensity values were averaged from multiple animals and standard deviations were calculated. A two-tail t-test was used to determine p-values.

For tibial and basitarsal segments of the prepupal legs, three squares were placed in the center of the *Scr* expression domain and equidistantly along the P/D axis. GFP intensity (pixels/cm) was measured with ImageJ from Z-projections. The same excitation settings and brightness/contrast image acquisition were used in all images that belong to the same quantification data set.

### Scr enhancer sequence identification and alignment

Genomic sequences homologous to the *D*. *melanogaster ScrE* sequence were analyzed via Blat using the *D*. *melanogaster* genome (BDGP release 6 + ISO1 MT/dm6) at the UCSC Genome Browser website.

## Supporting information

S1 Fig The *Scr* promoter directs low-level uniform expression and may be necessary for full enhancer activity. For all panels, proximal is left, and anterior is down. For panels A-B", Scr expression (anti-Scr) is shown in red and reporter expression is in green. A-A". Prepupal (6h APF) legs carrying the intronic enhancer in combination with the *hsp70* basal promoter (hs-intronic) compared to endogenous Scr expression. Note the weak and uneven GFP expression and the gap between the tibia (ti) and tarsal segment 1 (ta1). B-B". Prepupal (6h APF) legs carrying a GFP reporter gene under control of the *Scr* promoter compared to endogenous Scr expression. Note the low level uniform expression throughout the leg. C-C". Prepupal (6h APF) legs from animals bearing a GFP reporter gene under control of the upstream enhancer. Legs are from the T1 (C), T2 (C') or T3 (C") segments, identified via differential Ubx expression (anti-Ubx, red). T1 legs do not express Ubx (C), T2 legs have low-levels of Ubx expression in the posterior compartment and lack expression in the anterior compartment (C′). T3 legs have low-level Ubx expression in the anterior compartment and a strong expression in the posterior compartment (C″). GFP expression is observed all three legs and obeys the same boundaries along the A/P, D/V and P/D axes, but varies somewhat among segments due to the unique morphology of each leg.
(TIF)

S2 Fig The upstream *Scr* enhancer is not essential for development of T2 or T3 legs. In all leg images, proximal is up and the ventral surface is to the left. A. Female T2 legs homozygous for the CRISPR deletion of the upstream enhancer (*Scr*^*ΔCRE(upA-B)*^/ *Scr*^*ΔCRE(upA-B)*^). T2 legs exhibit a normal bristle pattern and no morphological changes are observed. B. Female T3 legs homozygous for the CRISPR deletion of the upstream enhancer (*Scr*^*ΔCRE(upA-B)*^/ *Scr*^*ΔCRE(upA-B)*^). T3 legs exhibit a normal morphology, including the presence of TBRs.
(TIF)

S3 Fig Summary of the upstream *Scr* enhancer analysis at three stages of leg development. A. Full set of fragment A sub-clones that were tested for enhancer activity. Black bars designate fragments that faithfully reproduce Scr expression in the TBR/SCB primordia; gray fragments direct no expression; red fragments direct altered spatial expression patterns compared to endogenous Scr expression; blue fragments direct lower levels of reporter expression compared to fragment A. Fragments that exhibit both reduced activity and direct altered spatial patterns are designated as both blue and red. B. Table summarizing expression data from all fragments, shown in panel A and Fig 3, that direct reporter gene expression in the TBR and/or SCB primordia. In the 3rd instar and prepupal (6h APF) stages, “F” designates full recapitulation of upregulated Scr expression, while F* designates fragments that direct normal spatial patterns of expression but for which expression level was not quantified. Fragments marked with “S” drove altered spatial expression, such as derepression of reporter expression in the posterior compartment or dorsal expansion of reporter expression, and for which expression level was not quantified. “I” designates fragments that directed lower levels of expression, and therefore did not fully recapitulate the upregulated Scr expression (S2 Table). I* designates fragments that direct visibly reduced expression but for which expression level was not quantified. In pupal legs (24h APF), endogenous Scr expression is upregulated around the presumptive sex comb, on both the proximo/dorsal and distal/ventral sides. Dark triangles designate fragments with correct expression, shaded gray triangles show fragments with weak or variable loss of GFP expression distal/ventral to the sex comb, and white triangles designate fragments with strong or consistent loss of reporter expression in the distal/ventral region.
(TIF)

S4 Fig Summary of reporter expression from various constructs in pupal legs. A-C. Reporter gene expression in the T1 legs of 24h pupae. All images show the anterior-ventral surface of the leg; ventral is left and anterior is right. The sex comb is originally specified as a single TBR at the distal tip of the ta1 segment; by this stage, it has almost finished clockwise rotation, as viewed from the ventral side, to assume a nearly longitudinal orientation along the PD leg axis. In all panels, GFP expression driven by reporter fragments is in green, and anti-Scr antibody staining is in red. A-A". Fragment A1 drives expression that recapitulates Scr pattern around the sex comb as well as in the more proximal TBRs (black bars in panel D). B-B". Fragment K shows partial loss of activity in a triangular region on the ventral (originally distal) side of the sex comb (arrow in B'). This loss of expression is minor and variable among individuals (dashed bars in panel D). C-C". Fragment E1 shows a stronger loss of expression in the ventral/distal triangle (arrow in C'), with little individual variation (grey bars in panel D). D-D". Expression driven by fragment D is weaker and less accurate than expression driven by the smaller fragment E1, which is completely encompassed by fragment D. E. Map of reporter fragments that show complete (black bars), weakly compromised (dashed bars), and strongly compromised (grey bars) expression in the sex comb region. Fragment E5 has no sex comb expression.
(TIF)

S5 Fig Fragments A and E exhibit comparable expression levels. A. Map of the *Scr* region (24). Intronic and upstream enhancers are shown relative to the *Scr* locus (dark blue lines designate exons, and light blue lines designate introns; introns are shown only for *Scr*). The intronic enhancer is located within the second intron of the *Scr* transcription unit, while the upstream enhancer is situated 33 kb 5’ of the *Scr* transcription start site. Three overlapping subfragments of the upstream 10 kb XbaI fragment, A, B and C were tested for enhancer activity, of which only A directed reporter expression in legs. Two Dorsal switch protein 1 (DSP1) binding sequences located within the 10 kb upstream enhancer are shown in gray relative to the upstream enhancer fragments (26). Black lines below the map designate regions to which Corto has been shown to bind in embryos (30), in which *Scr* expression is silenced in T2 and T3 segments. B. GFP signal quantification of identically imaged 3rd instar legs carrying either *A-GFP* or *ScrE-GFP* reporter (top). GFP intensity measurements taken from the domain of elevated Scr expression was divided by the background GFP intensity level to obtain normalized GFP values. The signal intensity in A-GFP expressing legs is slightly lower than in ScrE-GFP expressing legs (bottom) (*p = 0.0015). C. GFP signal quantification of identically imaged 6h APF prepupal legs carrying either the *A-GFP* or *ScrE-GFP* reporter (top). The signal intensity for A-GFP (blue bars) expressing legs is slightly lower than that for ScrE-GFP (green bars) expressing legs (bottom) (see S2 Table for statistics).
(TIF)

S6 Fig Putative En binding sites in CS6 function redundantly to inhibit posterior compartment expression of *Scr* A-A". A 3rd instar leg disc showing reporter expression (A′, yellow) from a transgenic line carrying fragment I, which lacks the CS6 sequence. Reporter expression is expanded to the posterior compartment (arrow) as compared to the endogenous Scr expression (A, magenta). B-B". A 3rd instar leg disc showing reporter expression from a transgenic line carrying *ScrE-GFP* with a mutation in the En-1 site (B′, green). Reporter expression was confined to the anterior compartment as compared to endogenous Scr expression (B, magenta). C-C". A 3rd instar leg disc showing reporter expression from a transgenic line carrying *ScrE-GFP* with a mutation in the En-2/3 site (C′, green). Reporter expression was confined to the anterior compartment as compared to the endogenous Scr expression (C, magenta).
(TIF)

S7 Fig A combination of Dll sites are required for elevated *Scr* expression. GFP intensity levels were measured in 3rd instar larval and 6h APF prepupal legs carrying various reporter genes, some of which have one or more mutation in putative Dll binding sites. Diagram of fragments tested with location of putative Dll binding sites is shown on the left. Mutated sites are marked with a red X. For prepupal legs, GFP intensity levels were measured in different regions along the P/D axis of the tibia and ta1 and averaged per segment (see Methods). Cells in dark gray show GFP intensity levels as a percentage of that of ScrE-GFP, which is arbitrarily set to 100%. Cells in light gray show GFP intensity level of E3.DllM1-GFP as a percentage of that of E3-GFP, which is arbitrarily set to 100%. NA: data not available. NS: no significant change. Data and statistics for quantifications of GFP intensity in larval and prepupal legs in this figure are shown in S2 Table. A. Fragment E contains three highly-conserved putative Dll binding sites and recapitulates the full spatial, temporal and intensity of *Scr* expression. B. Three Dll sites were mutated in the ScrE.DllM123-GFP reporter construct. ScrE.M123-GFP exhibits GFP intensity as low as 40.5% as compared to ScrE-GFP in larval leg discs. In 6h APF prepupal legs the GFP intensity average is 48.5% for the tibial and 52.2% for the ta1 segment (Fig 6). C. GFP intensity levels are lower in 3rd instar larval leg discs that carry the D-GFP reporter, which lacks sequences containing Dll-2 site. D. Reporter intensity is not reduced in 3rd instar leg discs when Dll-2 is mutated in isolation. However, GFP intensity from *ScrE*.*DllM2* is lower in prepupal legs, indicating that the Dll-2 is necessary for maintaining high levels of *Scr* expression. E. Two putative homeodomain binding sites (high-score Dll, En and Scr sites) were mutated in addition to the three Dll sites. Reporter intensity is 59.5% lower from ScrE.DllM123-GFP and 57.8% lower from ScrE.DllM12345-GFP compared to ScrE-GFP. F. E3-GFP is 53% lower in GFP intensity compared to ScrE-GFP. E3-GFP contains only Dll-1. G-H. E3.DllM1-GFP (H) intensity levels as compared to E3-GFP (G). Mutation of Dll-1 site in E3 results in further reduction in reporter intensity (41%).
(TIF)

S8 Fig Dll activates *ScrE* but not *Scr* expression in wing imaginal discs. A-A'". *Dll* gain-of-function clones in a 3rd instar larval wing are marked with mRFP expression (A, A'", magenta). ScrE-GFP expression (A', A'", green) is activated in the anterior compartment (marked by Ci expression, cyan in A", A'") but not in posterior compartment clones. B-B'". *Dll* gain-of-function clones in a 3rd instar larval wing are marked by mRFP expression (B, B'", magenta). ScrE.DllM123-GFP (B', B'", green) is activated in a subset of anterior compartment (marked by Ci expression cyan in B", B'"). Note that fewer clones express ScrE.DllM123-GFP as compared to ScrE-GFP expression in B-B'". C. *Dll* gain-of-function clones expressing ScrE-GFP or ScrE.DllM123-GFP were counted in multiple wings. ScrE-GFP expression was activated in 93.5% (43/46) of clones as compared to ScrE.DllM123-GFP which was expressed in 44% (32/73) of clones (*p =.0001).
(TIF)

S1 Table Separable elements direct low-level vs elevated Scr expression. The basal heat shock promoter and an *Scr* promoter fragment were tested in conjunction with the *Scr* intronic enhancer, and the *Scr* promoter was tested in combination with the upstream enhancer. All lines carrying transgenes that contain the *Scr* promoter direct low-level uniform reporter expression throughout the T1 leg, similar to the low-level expression pattern of Scr observed in T1 legs. On the other hand, the intronic enhancer specifically directs elevated expression in the TBR primordia when linked to either of the promoters tested. Both the intronic and upstream enhancers drive elevated expression in the TBR primordia, while upregulated expression around the SCB primordia is specifically driven by the upstream enhancer.
(TIF)

S2 Table Quantification of GFP signal intensity from a subset of the *Scr* upstream enhancer reporter genes. (XLSX)

S3 Table List of primers used in this study. (XLSX)
