# Supplementary figures and images for: A Distalless-responsive enhancer of the Hox gene Sex combs reduced is required for segment- and sex-specific sensory organ development in Drosophila
Source: PLoS Genet. 2018 Apr 10;14(4):e1007320. doi: 10.1371/journal.pgen.1007320 (PMC5909922; doi:10.1371/journal.pgen.1007320)

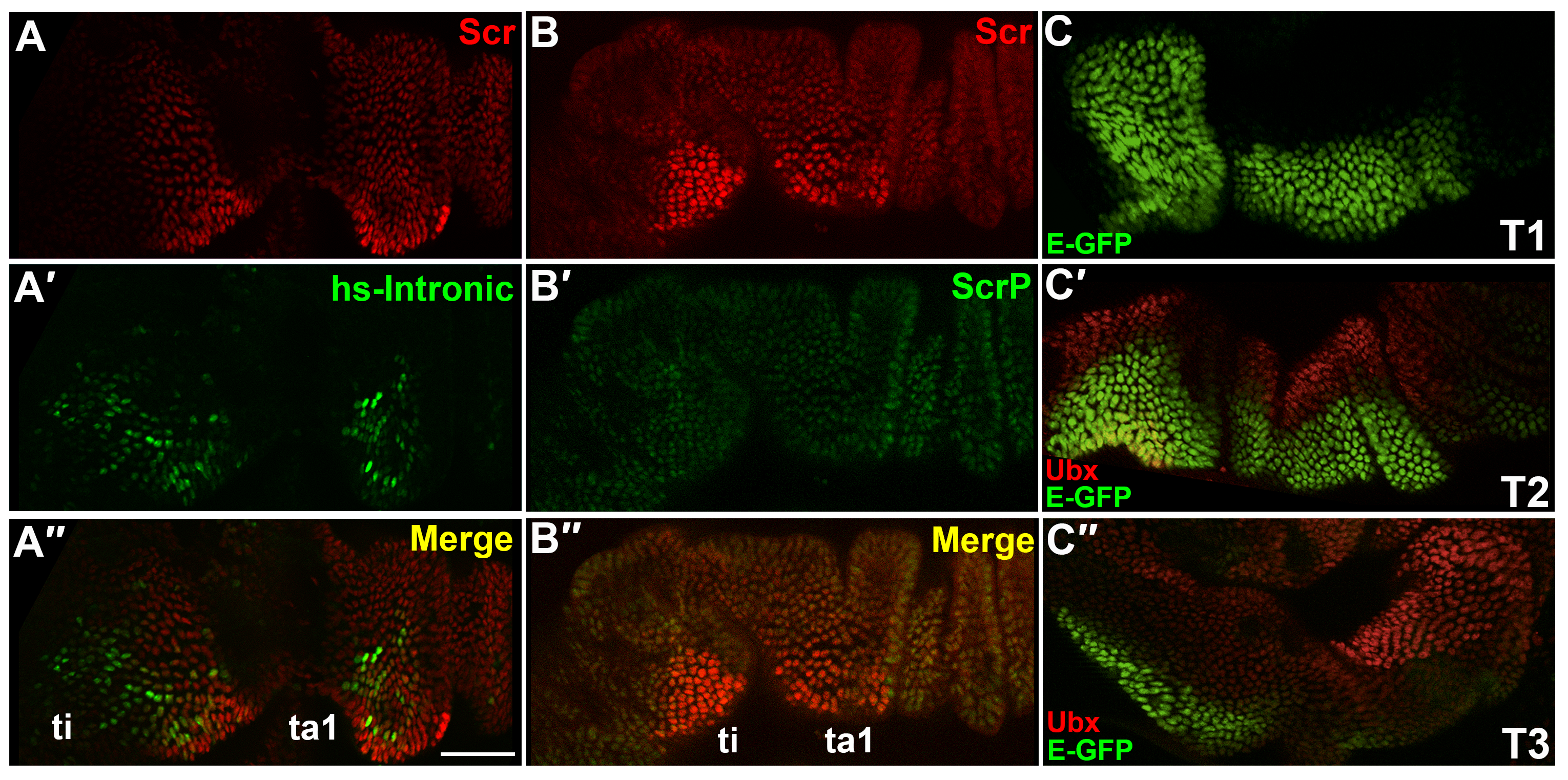

Supplement: S1 Fig — For all panels, proximal is left, and anterior is down. For panels A-B", Scr expression (anti-Scr) is shown in red and reporter expression is in green. A-A". Prepupal (6h APF) legs carrying the intronic enhancer in combination with the hsp70 basal promoter (hs-intronic) compared to endogenous Scr expression. Note the weak and uneven GFP expression and the gap between the tibia (ti) and tarsal segment 1 (ta1). B-B". Prepupal (6h APF) legs carrying a GFP reporter gene under control of the Scr promoter compared to endogenous Scr expression. Note the low level uniform expression throughout the leg. C-C". Prepupal (6h APF) legs from animals bearing a GFP reporter gene under control of the upstream enhancer. Legs are from the T1 (C), T2 (C') or T3 (C") segments, identified via differential Ubx expression (anti-Ubx, red). T1 legs do not express Ubx (C), T2 legs have low-levels of Ubx expression in the posterior compartment and lack expression in the anterior compartment (C′). T3 legs have low-level Ubx expression in the anterior compartment and a strong expression in the posterior compartment (C″). GFP expression is observed all three legs and obeys the same boundaries along the A/P, D/V and P/D axes, but varies somewhat among segments due to the unique morphology of each leg. (TIF) [file pgen.1007320.s001.tif]

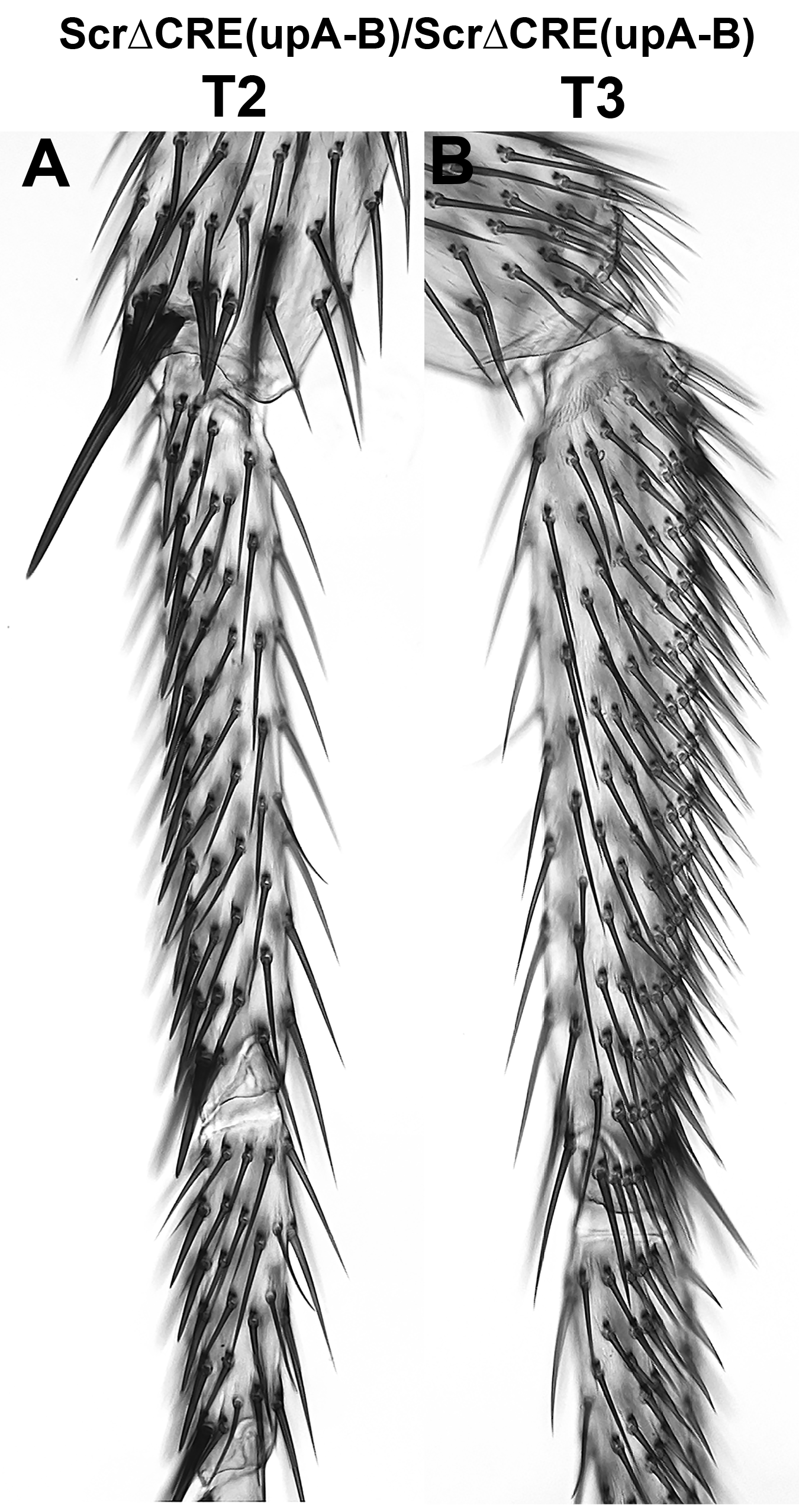

Supplement: S2 Fig — In all leg images, proximal is up and the ventral surface is to the left. A. Female T2 legs homozygous for the CRISPR deletion of the upstream enhancer (ScrΔCRE(upA-B)/ ScrΔCRE(upA-B)). T2 legs exhibit a normal bristle pattern and no morphological changes are observed. B. Female T3 legs homozygous for the CRISPR deletion of the upstream enhancer (ScrΔCRE(upA-B)/ ScrΔCRE(upA-B)). T3 legs exhibit a normal morphology, including the presence of TBRs. (TIF) [file pgen.1007320.s002.tif]

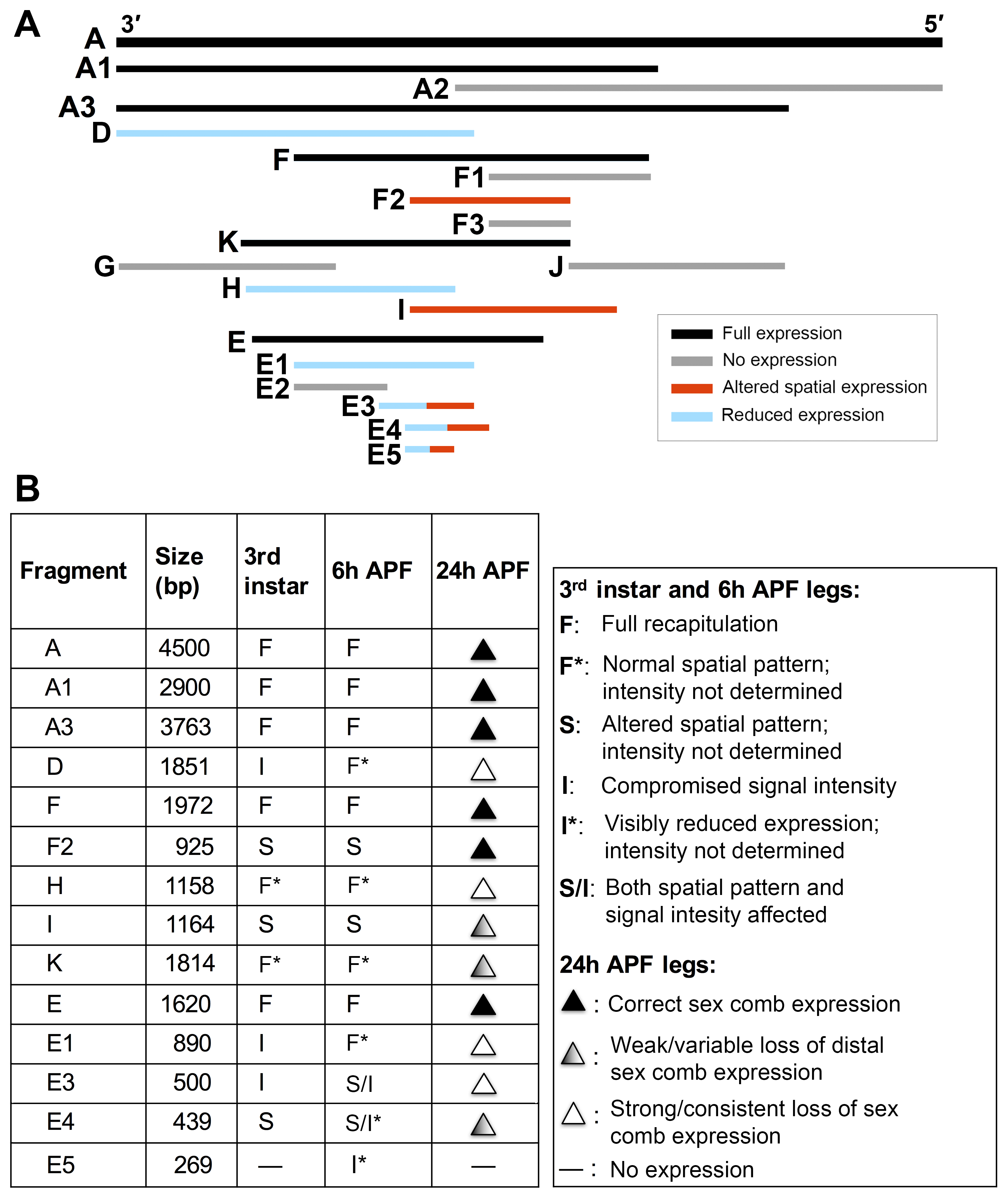

Supplement: S3 Fig — A. Full set of fragment A sub-clones that were tested for enhancer activity. Black bars designate fragments that faithfully reproduce Scr expression in the TBR/SCB primordia; gray fragments direct no expression; red fragments direct altered spatial expression patterns compared to endogenous Scr expression; blue fragments direct lower levels of reporter expression compared to fragment A. Fragments that exhibit both reduced activity and direct altered spatial patterns are designated as both blue and red. B. Table summarizing expression data from all fragments, shown in panel A and Fig 3, that direct reporter gene expression in the TBR and/or SCB primordia. In the 3rd instar and prepupal (6h APF) stages, “F” designates full recapitulation of upregulated Scr expression, while F* designates fragments that direct normal spatial patterns of expression but for which expression level was not quantified. Fragments marked with “S” drove altered spatial expression, such as derepression of reporter expression in the posterior compartment or dorsal expansion of reporter expression, and for which expression level was not quantified. “I” designates fragments that directed lower levels of expression, and therefore did not fully recapitulate the upregulated Scr expression (S2 Table). I* designates fragments that direct visibly reduced expression but for which expression level was not quantified. In pupal legs (24h APF), endogenous Scr expression is upregulated around the presumptive sex comb, on both the proximo/dorsal and distal/ventral sides. Dark triangles designate fragments with correct expression, shaded gray triangles show fragments with weak or variable loss of GFP expression distal/ventral to the sex comb, and white triangles designate fragments with strong or consistent loss of reporter expression in the distal/ventral region. (TIF) [file pgen.1007320.s003.tif]

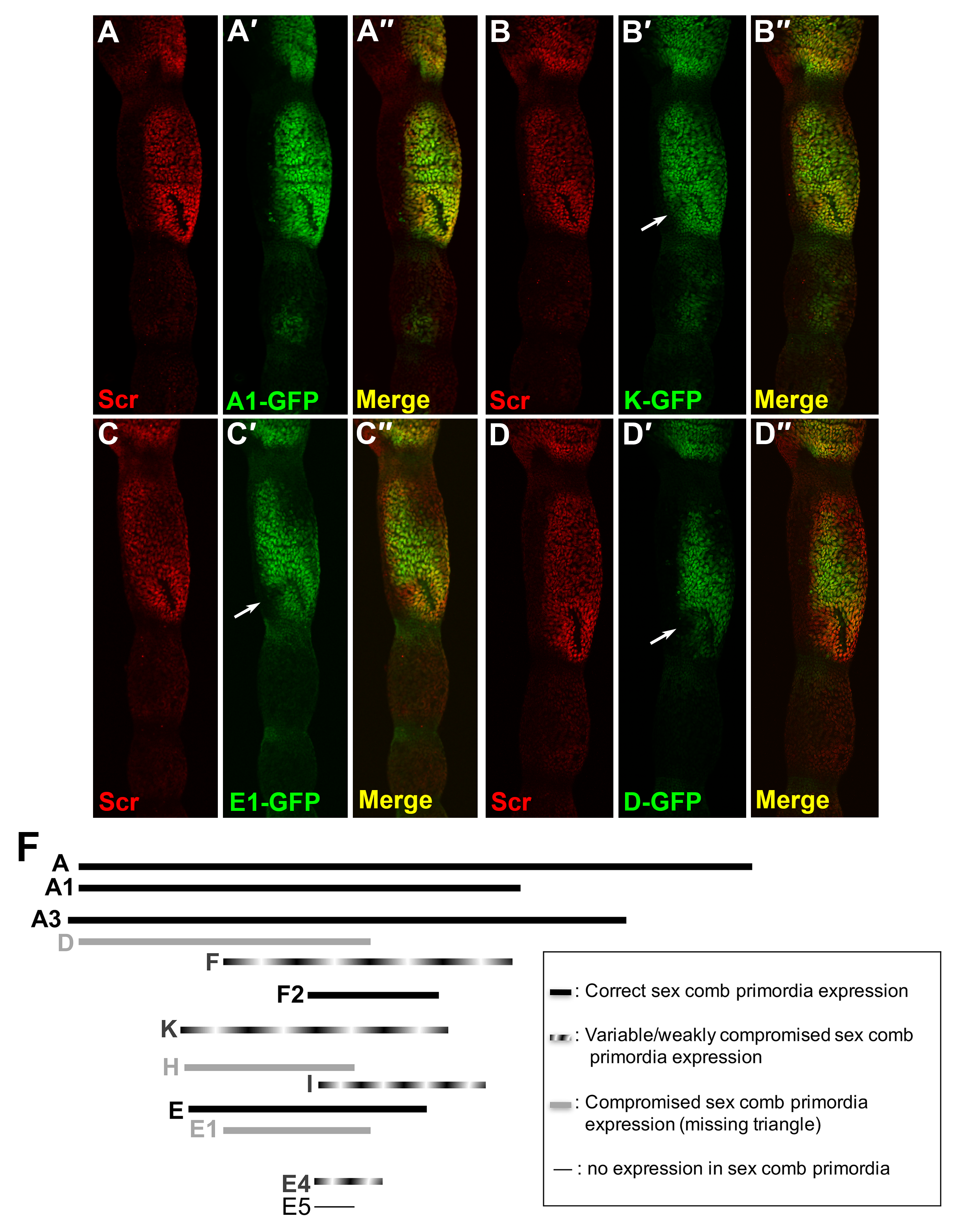

Supplement: S4 Fig — A-C. Reporter gene expression in the T1 legs of 24h pupae. All images show the anterior-ventral surface of the leg; ventral is left and anterior is right. The sex comb is originally specified as a single TBR at the distal tip of the ta1 segment; by this stage, it has almost finished clockwise rotation, as viewed from the ventral side, to assume a nearly longitudinal orientation along the PD leg axis. In all panels, GFP expression driven by reporter fragments is in green, and anti-Scr antibody staining is in red. A-A". Fragment A1 drives expression that recapitulates Scr pattern around the sex comb as well as in the more proximal TBRs (black bars in panel D). B-B". Fragment K shows partial loss of activity in a triangular region on the ventral (originally distal) side of the sex comb (arrow in B'). This loss of expression is minor and variable among individuals (dashed bars in panel D). C-C". Fragment E1 shows a stronger loss of expression in the ventral/distal triangle (arrow in C'), with little individual variation (grey bars in panel D). D-D". Expression driven by fragment D is weaker and less accurate than expression driven by the smaller fragment E1, which is completely encompassed by fragment D. E. Map of reporter fragments that show complete (black bars), weakly compromised (dashed bars), and strongly compromised (grey bars) expression in the sex comb region. Fragment E5 has no sex comb expression. (TIF) [file pgen.1007320.s004.tif]

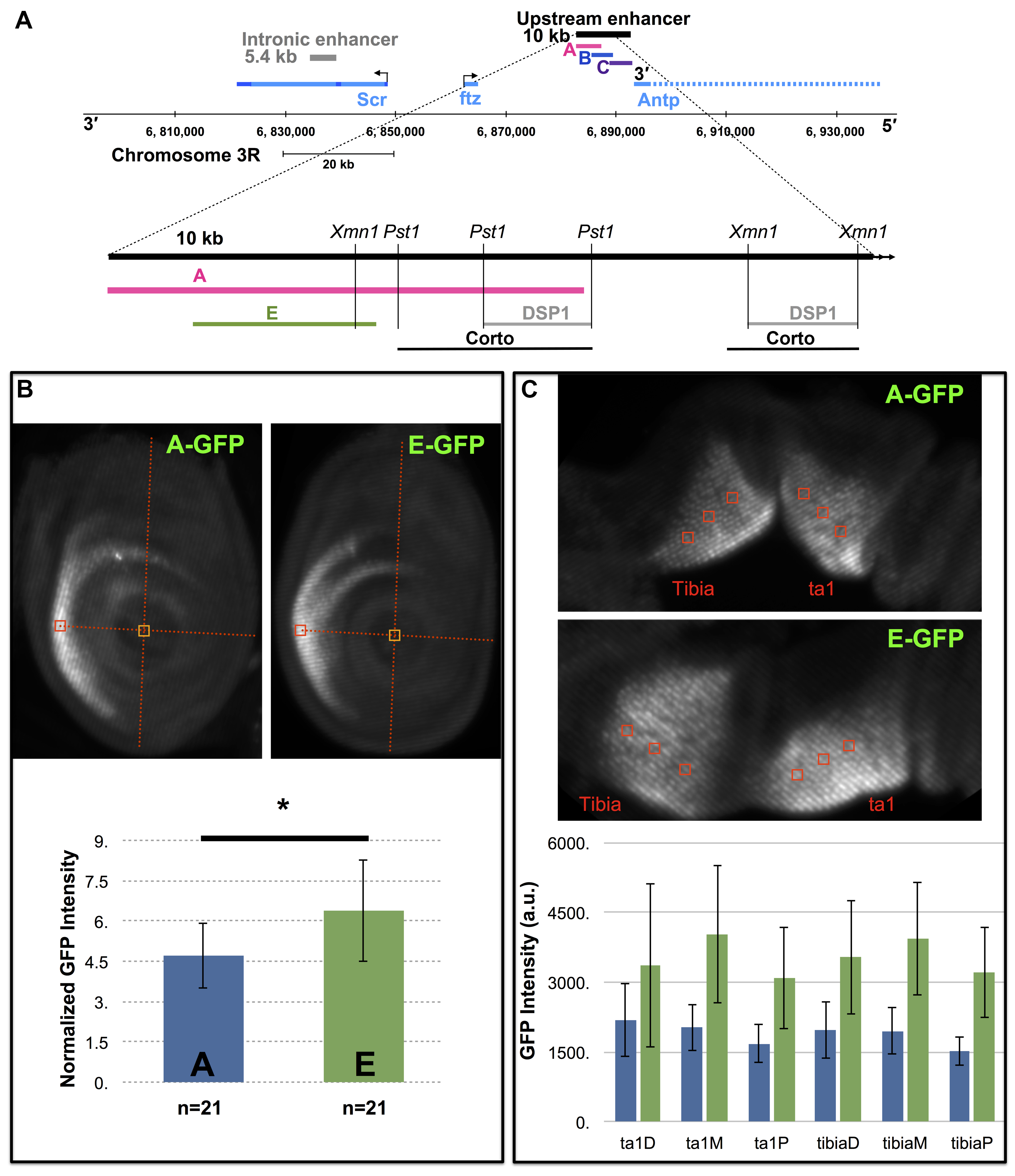

Supplement: S5 Fig — A. Map of the Scr region (24). Intronic and upstream enhancers are shown relative to the Scr locus (dark blue lines designate exons, and light blue lines designate introns; introns are shown only for Scr). The intronic enhancer is located within the second intron of the Scr transcription unit, while the upstream enhancer is situated 33 kb 5’ of the Scr transcription start site. Three overlapping subfragments of the upstream 10 kb XbaI fragment, A, B and C were tested for enhancer activity, of which only A directed reporter expression in legs. Two Dorsal switch protein 1 (DSP1) binding sequences located within the 10 kb upstream enhancer are shown in gray relative to the upstream enhancer fragments (26). Black lines below the map designate regions to which Corto has been shown to bind in embryos (30), in which Scr expression is silenced in T2 and T3 segments. B. GFP signal quantification of identically imaged 3rd instar legs carrying either A-GFP or ScrE-GFP reporter (top). GFP intensity measurements taken from the domain of elevated Scr expression was divided by the background GFP intensity level to obtain normalized GFP values. The signal intensity in A-GFP expressing legs is slightly lower than in ScrE-GFP expressing legs (bottom) (*p = 0.0015). C. GFP signal quantification of identically imaged 6h APF prepupal legs carrying either the A-GFP or ScrE-GFP reporter (top). The signal intensity for A-GFP (blue bars) expressing legs is slightly lower than that for ScrE-GFP (green bars) expressing legs (bottom) (see S2 Table for statistics). (TIF) [file pgen.1007320.s005.tif]

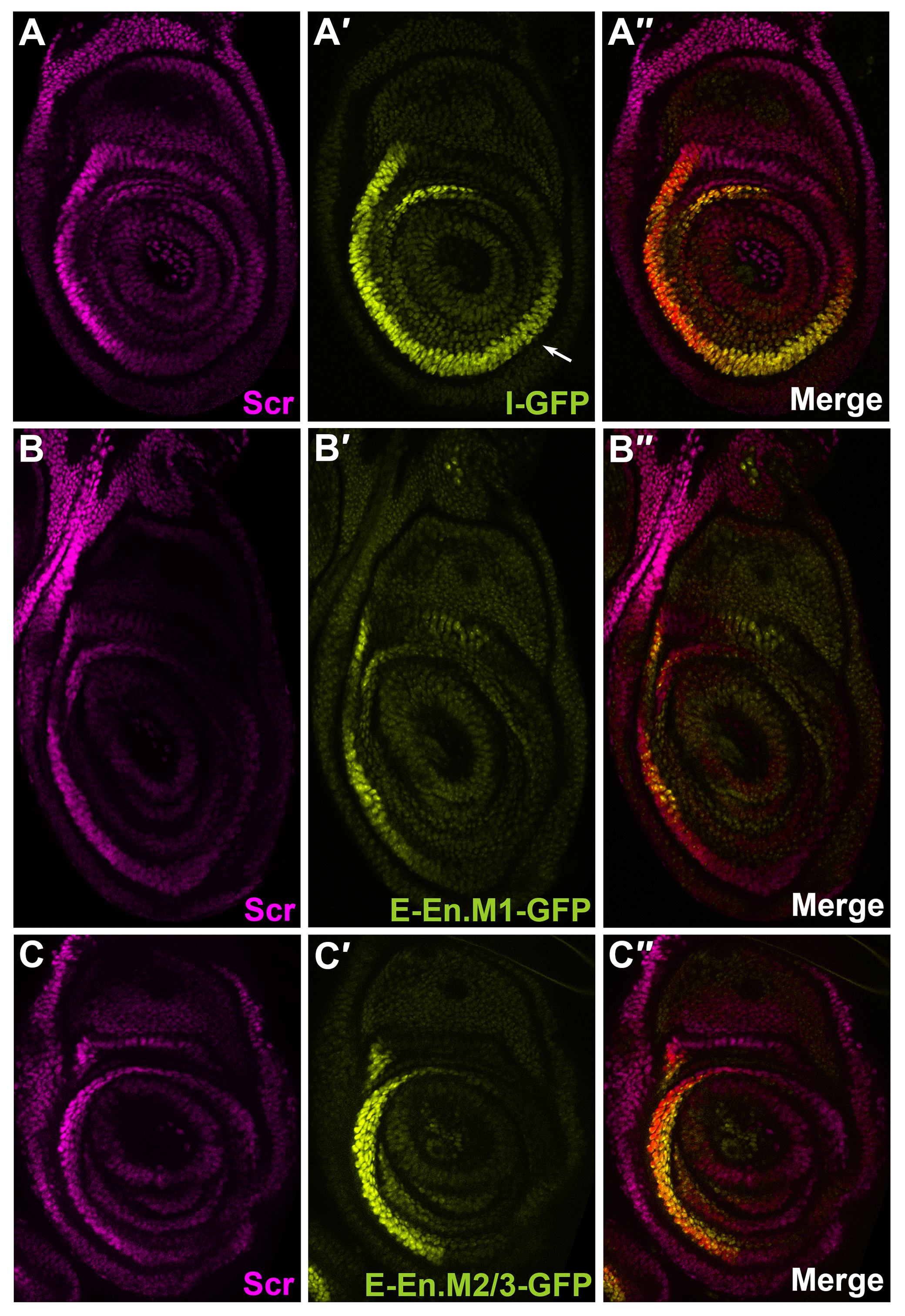

Supplement: S6 Fig — A 3rd instar leg disc showing reporter expression (A′, yellow) from a transgenic line carrying fragment I, which lacks the CS6 sequence. Reporter expression is expanded to the posterior compartment (arrow) as compared to the endogenous Scr expression (A, magenta). B-B". A 3rd instar leg disc showing reporter expression from a transgenic line carrying ScrE-GFP with a mutation in the En-1 site (B′, green). Reporter expression was confined to the anterior compartment as compared to endogenous Scr expression (B, magenta). C-C". A 3rd instar leg disc showing reporter expression from a transgenic line carrying ScrE-GFP with a mutation in the En-2/3 site (C′, green). Reporter expression was confined to the anterior compartment as compared to the endogenous Scr expression (C, magenta). (TIF) [file pgen.1007320.s006.tif]

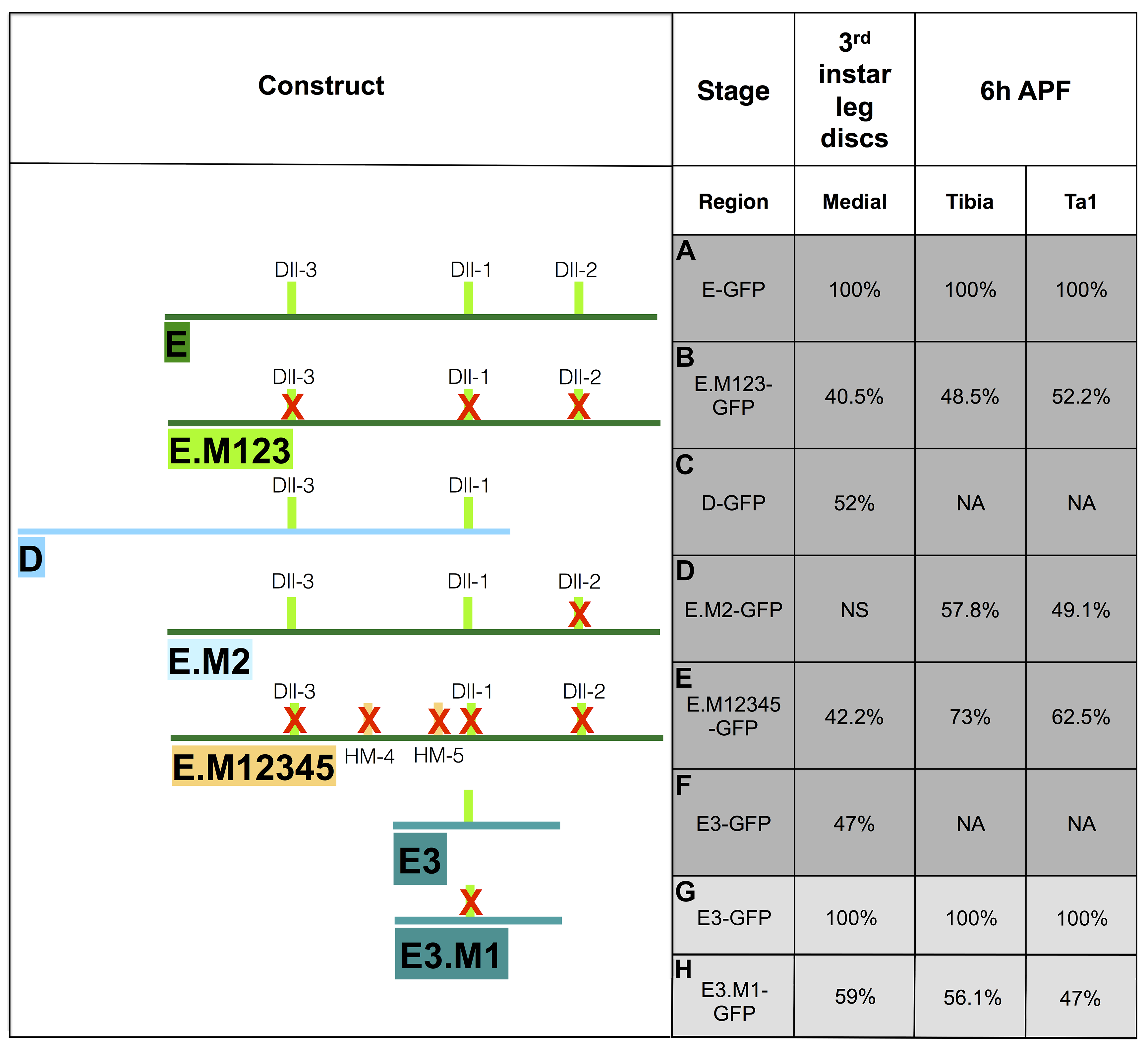

Supplement: S7 Fig — GFP intensity levels were measured in 3rd instar larval and 6h APF prepupal legs carrying various reporter genes, some of which have one or more mutation in putative Dll binding sites. Diagram of fragments tested with location of putative Dll binding sites is shown on the left. Mutated sites are marked with a red X. For prepupal legs, GFP intensity levels were measured in different regions along the P/D axis of the tibia and ta1 and averaged per segment (see Methods). Cells in dark gray show GFP intensity levels as a percentage of that of ScrE-GFP, which is arbitrarily set to 100%. Cells in light gray show GFP intensity level of E3.DllM1-GFP as a percentage of that of E3-GFP, which is arbitrarily set to 100%. NA: data not available. NS: no significant change. Data and statistics for quantifications of GFP intensity in larval and prepupal legs in this figure are shown in S2 Table. A. Fragment E contains three highly-conserved putative Dll binding sites and recapitulates the full spatial, temporal and intensity of Scr expression. B. Three Dll sites were mutated in the ScrE.DllM123-GFP reporter construct. ScrE.M123-GFP exhibits GFP intensity as low as 40.5% as compared to ScrE-GFP in larval leg discs. In 6h APF prepupal legs the GFP intensity average is 48.5% for the tibial and 52.2% for the ta1 segment (Fig 6). C. GFP intensity levels are lower in 3rd instar larval leg discs that carry the D-GFP reporter, which lacks sequences containing Dll-2 site. D. Reporter intensity is not reduced in 3rd instar leg discs when Dll-2 is mutated in isolation. However, GFP intensity from ScrE.DllM2 is lower in prepupal legs, indicating that the Dll-2 is necessary for maintaining high levels of Scr expression. E. Two putative homeodomain binding sites (high-score Dll, En and Scr sites) were mutated in addition to the three Dll sites. Reporter intensity is 59.5% lower from ScrE.DllM123-GFP and 57.8% lower from ScrE.DllM12345-GFP compared to ScrE-GFP. F. E3-GFP is 53% lower in GFP inten [file pgen.1007320.s007.tif]

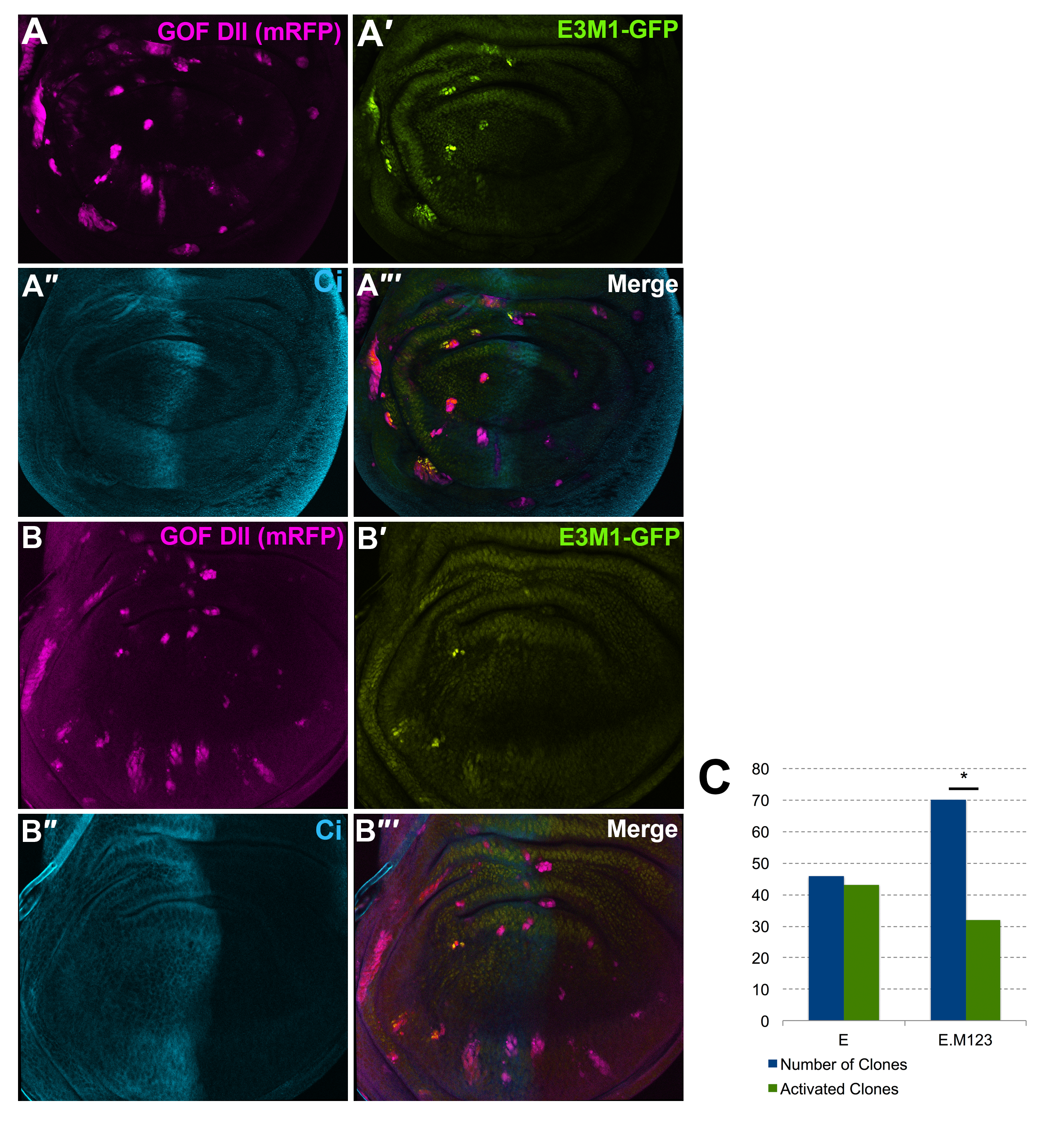

Supplement: S8 Fig — A-A'". Dll gain-of-function clones in a 3rd instar larval wing are marked with mRFP expression (A, A'", magenta). ScrE-GFP expression (A', A'", green) is activated in the anterior compartment (marked by Ci expression, cyan in A", A'") but not in posterior compartment clones. B-B'". Dll gain-of-function clones in a 3rd instar larval wing are marked by mRFP expression (B, B'", magenta). ScrE.DllM123-GFP (B', B'", green) is activated in a subset of anterior compartment (marked by Ci expression cyan in B", B'"). Note that fewer clones express ScrE.DllM123-GFP as compared to ScrE-GFP expression in B-B'". C. Dll gain-of-function clones expressing ScrE-GFP or ScrE.DllM123-GFP were counted in multiple wings. ScrE-GFP expression was activated in 93.5% (43/46) of clones as compared to ScrE.DllM123-GFP which was expressed in 44% (32/73) of clones (*p =.0001). (TIF) [file pgen.1007320.s008.tif]

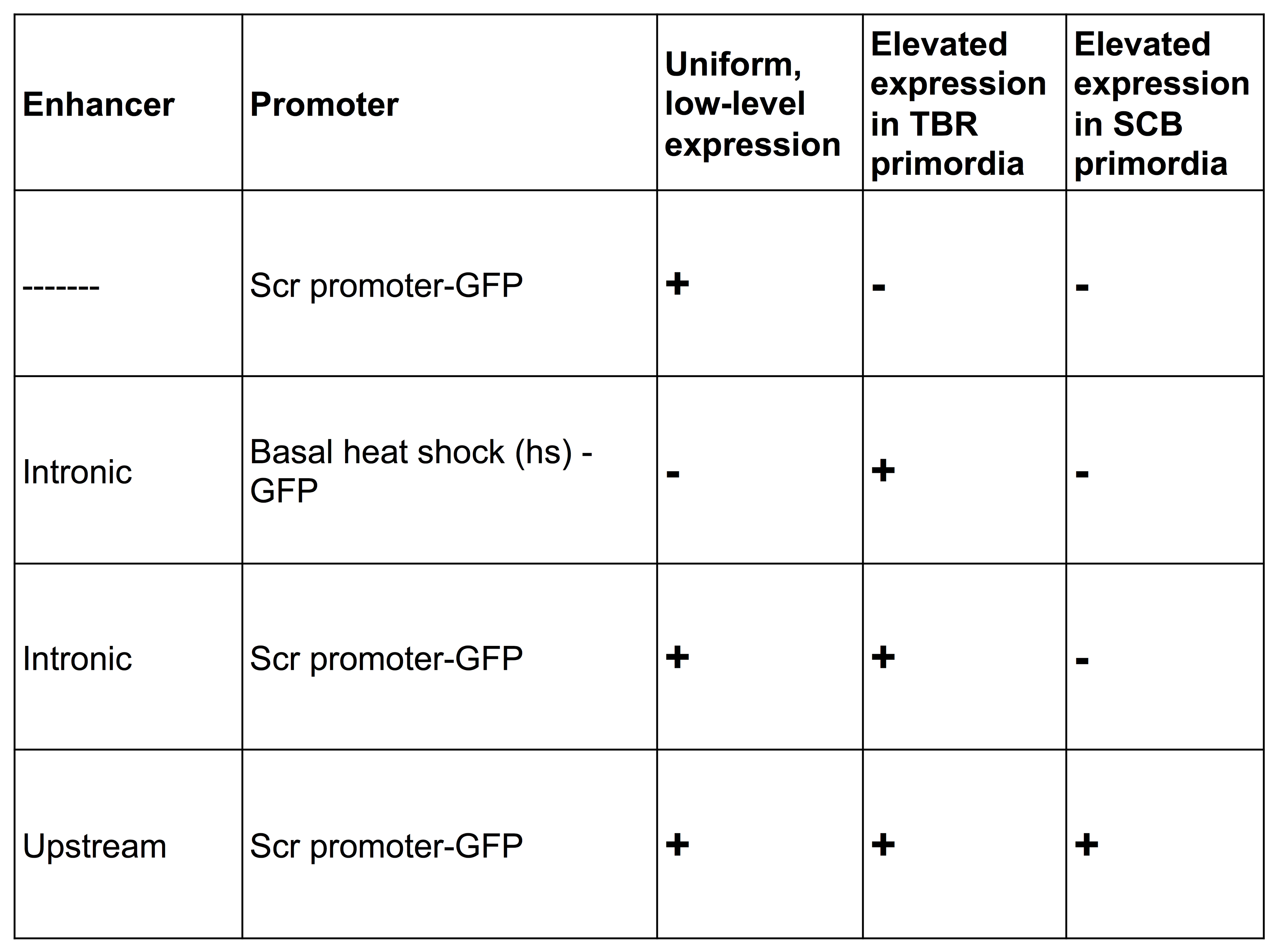

Supplement: S1 Table — The basal heat shock promoter and an Scr promoter fragment were tested in conjunction with the Scr intronic enhancer, and the Scr promoter was tested in combination with the upstream enhancer. All lines carrying transgenes that contain the Scr promoter direct low-level uniform reporter expression throughout the T1 leg, similar to the low-level expression pattern of Scr observed in T1 legs. On the other hand, the intronic enhancer specifically directs elevated expression in the TBR primordia when linked to either of the promoters tested. Both the intronic and upstream enhancers drive elevated expression in the TBR primordia, while upregulated expression around the SCB primordia is specifically driven by the upstream enhancer. (TIF) [file pgen.1007320.s009.tif]
